# A four-part working bibliography of neuroethics: Part 4 - Ethical issues in clinical and social applications of neuroscience

**DOI:** 10.1186/s13010-017-0043-y

**Published:** 2017-05-31

**Authors:** Kira Becker, John R. Shook, Martina Darragh, James Giordano

**Affiliations:** 1 Department of Neuroscience, Amherst College, Amherst MA, USA; 2 Department of Philosophy, University at Buffalo, Buffalo, NY USA; 3 Bioethics Research Library, Kennedy Institute of Ethics, Georgetown University,, Washington, DC, USA; 4 Neuroethics Studies Program, Pellegrino Center for Clinical Bioethics, and Department of Neurology, Georgetown University Medical Center, Washingotn, DC, USA

**Keywords:** Neuroethics, Neuroscience, Neurology, Psychiatry, Bibliography

## Abstract

**Background:**

As a discipline, neuroethics addresses a range of questions and issues generated by basic neuroscientific research (inclusive of studies of putative neurobiological processes involved in moral and ethical cognition and behavior), and its use and meanings in the clinical and social spheres. Here, we present Part 4 of a four-part bibliography of the neuroethics literature focusing on clinical and social applications of neuroscience, to include: the treatment-enhancement discourse; issues arising in neurology, psychiatry, and pain care; neuroethics education and training; neuroethics and the law; neuroethics and policy and political issues; international neuroethics; and discourses addressing "trans-" and "post-" humanity.

**Methods:**

To complete a systematic survey of the literature, 19 databases and 4 individual open-access journals were employed. Searches were conducted using the indexing language of the U.S. National Library of Medicine (NLM). A Python code was used to eliminate duplications in the final bibliography.

**Results:**

When taken with Parts 1-3, this bibliography aims to provide a listing of international peerreviewed papers, books, and book chapters published from 2002 through 2016. While seeking to be as comprehensive as possible, it may be that some works were inadvertently and unintentionally not included. We therefore invite commentary from the field to afford completeness and contribute to this bibliography as a participatory work-in-progress.

## Introduction

In *A Theory of Justice*, philosopher John Rawls proposed that the ethico-legal structure of society is based those ways that constructs of rightness or wrongness are applied to any situation [1]. The citations in this fourth part of a bibliography of neuroethics reflect works that focus upon the social aspects of research and clinical advances in the brain sciences. To be sure, neuroscience is not confined to the laboratory, as the demand for, and concomitant concerns about “bench-to-bedside” translation are increasing. Questions about the use of neuroscientific approaches to define normality; the meaning and relevance of what constitutes (and who receives) treatment or enhancement; the ability and validity of using neuroimaging to depict consciousness; and the trajectory of human biology and society, each and all arise from the interfluence of neuroscientific advancement and social expectation(s) and anxieties. Addressing these questions is, and will not be, simple or easy. As Illes et al. have noted, “…frontier technology that is able to touch on our personhood, especially in bioscience and information science, is shaping our future” [2].

The direction of brain science can, and arguably should be guided by neuroethics. The neuroethics literature “…seeks to give neuroscience what bioethics and the ethical, legal and social implication (ELSI) programs provided for the human genome project: a platform for scientists, lawyers, philosophers, sociologists, other scholars and the general public to interact and discuss the future of neuroscience” [3]. In this way, the literature – and this bibliography – provide a view to the discourse to date, and the foundation upon which to build engagement at present and in the future. To be sure, this future will ever more involve, and affect the world stage, as brain science becomes an increasingly international enterprise. Viewed from an international vantage point, neuroethics has the potential to go beyond “…absolutism, cultural essentialisms, and unrealistic ethical philosophies, [and] arrives at a small set of principles about proper human flourishing that are more culturally inclusive and cosmopolitan in spirit…empowerment, non-obsolescence, self-creativity, and citizenship” [4].

The inclusion of “citizenship” is important, because both professionals (in the natural, physical, life and social sciences, medicine, and government) and various publics “…must have the power — defined by quality of knowledge and ease of access — to help shape that future”. … It is in this spirit that a consistent goal of this bibliographic series is to invite ongoing professional and public participation in contributing to its further development, and in so doing bolstering the informational groundwork upon which the future of neuroscience can stand.

The following bibliographies provide neuroethics’ literature on the clinical and social implications of neurological advances from 2002–2016. The bibliographies cover a range of topics, to include:

I. Health care
  - issues in/of the treatment-enhancement discourse
  - ethical issues in neurology (including neuro-rehabilitation)
  - ethical issues in psychiatry (including gender and LGBT issues)
  - ethical issues in anesthesiology/pain medicine (including addiction)
II. Neuroethics education/training
III. Neuroethics and law
IV. Neuroethics and policy and politics
V. International neuroethics
VI. “Trans”/“post” human issues

Methods for systematically searching relevant literature devoted to neuroethics are identical to those utilized in the first 3 parts of this bibliography [5–7]. The United States National Library of Medicine’s (NLM) indexing language–MeSH (Medical Subject Headings)–was used to generate the basic search strategy for each topic. MeSH contains ethics-related terms developed for BIOETHICSLINE, a specialty database devoted to bioethical issues produced for NLM by the Kennedy Institute of Ethics from 1975–2000.

Citations were retrieved from the following databases:

1. U.S. National Library of Medicine’s PubMed and NLM Catalog
2. Academic Search Premier
3. Proquest Research Library
4. JSTOR
5. WorldCat
6. Philosopher’s Index
7. Embase
8. BELIT
9. Web of Knowledge/Web of Science
10. Digital Public Library of America
11. Directory of Open Access Journals
12. Hathi Trust Digital Library
13. Internet Archive
14. Globethics.net
15. Neuroethics-Wikiography

These listings of citations are “participatory bibliographies” in that readers are encouraged to submit additional cites via the “Comments” section of this document or by contacting the bibliographic manager at bioethics@georgetown.edu.

## Health Care

### Issues in/of the treatment-enhancement discourse

**Articles:**

Alberini CM, Chen DY: **Memory enhancement: consolidation, reconsolidation and insulin-like growth factor 2.** *Trends Neurosci* 2012, **35**(5):274-283. doi: 10.1016/j.tins.2011.12.007.

Asscher EC, Schermer M: **Wish-fulfilling medicine in practice: the opinions and arguments of lay people.** *J Med Ethics* 2014, **40**(12):837-841. doi:10.1136/medethics-2013-101480.

Attiah MA, Farah MJ: **Minds, motherboards, and money: futurism and realism in the neuroethics of BCI technologies**. *Front Syst Neurosci* 2014, **8**:86. doi:10.3389/fnsys.2014.00086.

Bamford R: **Unrequited: neurochemical enhancement of love**. *Camb Q Healthc Ethics* 2015, **24**(3):355-360. doi:10.1017/S0963180114000668.

Benanti P: **Neuroenhancement in young people: cultural need or medical technology?** *AJOB Neurosci* 2010, **1**(1):27-29. doi:10.1080/21507740903523210.

Beyer C, Staunton C, Moodley K: **The implications of methylphenidate use by healthy medical students and doctors in South Africa.** *BMC Med Ethics* 2014, **15**:20. doi:10.1186/1472-6939-15-20.

Boldt J: **[Doping, enhancement, betterment: tasks of tomorrow’s medicine?].** *Dtsch Med Wochenschr* 2010, **135**(37):1823-1826. doi:10.1055/s-0030-1263324.

Bolt LL: **True to oneself? broad and narrow ideas on authenticity in the enhancement debate**. *Theor Med Bioeth* 2007, **28**(4):285-300. doi:10.1007/s11017-007-9039-8.

Bostrom N, Sandberg A: **Cognitive enhancement: methods, ethics, regulatory challenges**. *Sci Eng Ethics* 2009, **15**(3):311-341. doi:10.1007/s11948-009-9142-5.

Brewer CD, DeGrote H: **Regulating methylphenidate: enhancing cognition and social inequality**. *Am J Bioeth* 2013, **13**(7):47-49. doi:10.1080/15265161.2013.794886.

Brühl AB, Sahakian BJ: **Drugs, games, and devices for enhancing cognition: implications for work and society.** *Ann N Y Acad Sci* 2016, **1369**(1):195-217. doi:10.1111/nyas.13040.

Bruhn C: **[Neuroenhancement: doping for the brain].** *Dtsch Med Wochenschr* 2009, **134**(40): 35. doi:10.1055/s-0028-1124088.

Busardò FP et al.: **From clinical application to cognitive enhancement: the example of methylphenidate.** *Curr Neuropharmacol* 2016, **14**(1):17-27. doi: 10.2174/1570159X13666150407225902.

Cabrera L, Weckert J: **Human enhancement and communication: on meaning and shared understanding.** *Sci Eng Ethics* 2013, **19**(3):1039-1056. doi:10.1007/s11948-012-9395-2.

Cabrera LY, Elger BS: **Memory interventions in the criminal justice system: some practical ethical considerations.** *J Bioeth Inq* 2016, **13**(1):95-103. doi:10.1007/s11673-015-9680-2.

Cabrera LY, Reiner PB: **Understanding public (mis)understanding of tDCS for enhancement.** *Front Integr Neurosci* 2015, **9**:30. doi:10.3389/fnint.2015.00030.

Caplan AL: **Important treatment, wrong diagnosis: enhancement is not to blame for the abuse of autonomy**. *Cerebrum* 2004, **6**(4):25-26.

Caplan AL, McHugh PR: **Shall we enhance? a debate.** *Cerebrum* 2004, **6**(4):13-29.

Clausen J: **Conceptual and ethical issues with brain-hardware interfaces.** *Curr Opin Psychiatry* 2011, **24**(6):495-501. doi:10.1097/YCO.0b013e32834bb8ca.

Cooze J, Gillam L: **Democratizing “psychotropic neuroenhancement.”** *AJOB Neurosci* 2010, **1**(1):19-20. doi:10.1080/21507740903504491.

Danaher J: **An evaluative conservative case for biomedical enhancement**. *J Med Ethics* 2016, **42**(9):611-618. doi:10.1136/medethics-2015-103307.

de Jongh R, Bolt I, Schermer M, Olivier B: **Botox for the brain: enhancement of cognition, mood and pro-social behavior and blunting of unwanted memories.** *Neurosci Biobehav Rev* 2008, **32**(4):760-776. doi:10.1016/j.neubiorev.2007.12.001.

Dietz P, Soyka M, Franke AG: **Pharmacological neuroenhancement in the field of economics-poll results from an online survey.** *Front Psychol* 2016, **7**:520. doi:10.3389/fpsyg.2016.00520.

Dubljević V: **Prohibition or coffee shops: regulation of amphetamine and methylphenidate for enhancement use by healthy adults**. *Am J Bioeth* 2013, **13**(7):23-33. doi:10.1080/15265161.2013.794875.

Dubljević V: **Response to open peer commentaries on "prohibition or coffee shops: regulation of amphetamine and methylphenidate for enhancement use by healthy adults".** *Am J Bioeth* 2014, **14**(1):W1-W8. doi:10.1080/15265161.2014.862417.

Earp BD, Sandberg A, Kahane G, Savulescu J: **When is diminishment a form of enhancement? Rethinking the enhancement debate in biomedical ethics**. *Front Syst Neurosci* 2014, **8**:12. doi:10.3389/fnsys.2014.00012.

Eberl JT: **A Thomistic appraisal of human enhancement technologies.** *Theor Med Bioeth* 2014, **35**(4):289-310. doi:10.1007/s11017-014-9300-x.

Eßmann B: **Human enhancement: revisiting the ethical framework**. *Med Health Care Philos* 2011, **14**(4):425-427. doi:10.1007/s11019-011-9350-z.

Erler A: **Does memory modification threaten our authenticity?** *Neuroethics* 2011, **4**(3): 235-249. doi:10.1007/s12152-010-9090-4.

Faber NS, Savulescu J, Douglas T: **Why is cognitive enhancement deemed unacceptable? the role of fairness, deservingness, and hollow achievements**. *Front Psychol* 2016, **7**:232. doi: 10.3389/fpsyg.2016.00232.

Farah MJ, Smith ME, Ilieva I, Hamilton RH: **Cognitive enhancement**. *Wiley Interdiscip Rev Cogn Sci* 2014, **5**(1):95-103. doi:10.1002/wcs.1250.

Farah MJ et al.: **Neurocognitive enhancement: what can we do and what should we do?** *Nat Rev Neurosci* 2004, **5**(5):421-425. doi:10.1038/nrn1390.

Farah MJ: **The unknowns of cognitive enhancement**. *Science* 2015, **350**(6259):379-380. doi:10.1126/science.aad5893.

Faulmüller N, Maslen H, de Sio FS: **The indirect psychological costs of cognitive enhancement**. *Am J Bioeth* 2013, **13**(7):45-47. doi:10.1080/15265161.2013.794880.

Fenton A: **Buddhism and neuroethics: the ethics of pharmaceutical cognitive enhancement**. *Dev World Bioeth* 2009, **9**(2):47-56. doi:10.1111/j.1471-8847.2007.00226.x.

Fleischman A et al.: **Dealing with the long-term social implications of research.** *Am J Bioeth* 2011, **11**(5):5-9. doi:10.1080/15265161.2011.568576.

Flower K et al.: **Efficacy, safety, and ethics of cosmetic neurology far from settled.** *Clin Pharmacol Ther* 2010, **88**(4):461-463. doi:10.1038/clpt.2010.194.

Fond G et al.: **Innovative mechanisms of action for pharmaceutical cognitive enhancement: a systematic review.** *Psychiatry Res* 2015, **229**(1-2):12-20. doi:10.1016/j.psychres.2015.07.006.

Fond G et al.: **(Mis)use of prescribed stimulants in the medical student community: motives and behaviors: a population-based cross-sectional study.** *Medicine (Baltimore)* 2016, **95** (16):e3366. doi:10.1097/MD.0000000000003366.

Forlini C, Racine E: **Considering the causes and implications of ambivalence in using medicine for enhancement.** *Am J Bioeth* 2011, **11**(1):15-17. doi: 10.1080/15265161.2011.534952.

Forlini C, Racine E, Vollmann J, Schildmann J: **How research on stakeholder perspectives can inform policy on cognitive enhancement.** *Am J Bioeth* 2013, **13**(7):41-43. doi:10.1080/15265161.2013.794882.

Forlini C et al.: **Navigating the enhancement landscape: ethical issues in research on cognitive enhancers for healthy individuals.** *EMBO Rep* 2013, **14**(2):123-128. doi:10.1038/embor.2012.225.

Forlini C, Racine E: **Stakeholder perspectives and reactions to “academic” cognitive enhancement: unsuspected meaning of ambivalence and analogies.** *Public Underst Sci* 2012, **21**(5):606-625. doi:10.1177/0963662510385062.

Franke AG, Northoff R, Hildt E: **The case of pharmacological neuroenhancement: medical, judicial and ethical aspects from a German perspective.** *Pharmacopsychiarty* 2015, **48**(7): 256-264. doi:10.1055/s-0035-1559640.

Franke AG et al.: **[Characteristics of university students using stimulants for cognitive enhancement: a pilot study].** *Psychiatr Prax* 2012, **39**(4):174-180. doi: 10.1055/s-0031-1298900.

Franke AG, Soyka M: **[Pharmacological cognitive enhancement from a perspective of misuse and addiction].** *Fortschr Neurol Psychiatr* 2015, **83**(2):83-90. doi: 10.1055/s-0034-1398935.

Franke AG, Lieb K: **[Pharmacological neuroenhancement and brain doping: chances and risks].** *Bundesgesundheitsblatt Gesundheitsforschung Gesundheitsschutz* 2010, **53**(8):853-859. doi:10.1007/s00103-010-1105-0.

Frati P et al.: **Smart drugs and synthetic androgens for cognitive and physical enhancement: revolving doors of cosmetic neurology.** *Curr Neuropharmacol* 2015, **13**(1):5-11. doi:10.2174/1570159X13666141210221750.

Garasic MD, Lavazza A: **Moral and social reasons to acknowledge the use of cognitive enhancers in competitive-selective contexts.** *BMC Med Ethics* 2016, **17**:18. doi:10.1186/s12910-016-0102-8.

Gini A, Rossi J, Giordano J: **Considering enhancement (and/or treatment): on the need to regard contingency and develop dialectic evaluation**. *AJOB Neurosci* 2010,**1**(1):25-27. doi:10.1080/21507740903504392.

Glannon W: **Altering the brain and mind**. *Hastings Cent Rep* 2008, **38**(4):46-47. doi:10.1353/hcr.0.0039.

Glannon W: **Psychopharmacological enhancement**. *Neuroethics* 2008, **1**(1):45-54. doi: 10.1007/s12152-008-9005-9.

Gleaves J: **A new conceptual gloss that still lacks luster: critiquing Morgan's treatment-enhancement distinction**. *J Philos Sport* 2011, **38**(1):103-112. doi:10.1080/00948705.2011.9714552.

Greely HT: **Knowing sin: making sure good science doesn’t go bad**. *Cerebrum* 2006, 1-8.

Greely HT: **Some first steps toward responsible use of cognitive-enhancing drugs by the healthy**. *Am J Bioeth* 2013, **13**(7):39-41. doi:10.1080/15265161.2013.795823.

Hall W, Partridge B, Lucke J: **Constraints on regulatory options for putatively cognitive enhancing drugs**. *Am J Bioeth* 2013, **13**(7):35-37. doi:10.1080/15265161.2013.795825.

Hall WD, Lucke JC: **The enhancement use of neuropharmaceuticals: more scepticism and caution needed.** *Addiction* 2010, **105**(12):2041-2043. doi:10.1111/j.1360-0443.2010.03211.x.

Holtug N: **Equality and the treatment-enhancement distinction**. *Bioethics* 2011, **25**(3):137-144. doi:10.1111/j.1467-8519.2009.01750.x.

Huggins J, Simmerling M: **Normal functioning and the treatment/enhancement distinction: an opportunity based assessment**. *J Relig Health* 2014, **53**(4):1214-1222. doi:10.1007/s10943-014-9882-7.

Hyman SE: **Cognitive enhancement: promises and perils**. *Neuron* 2011, **69**(4):595-598. doi:10.1016/j.neuron.2011.02.012.

Jain KK: **Future of nanomedicine: impact on healthcare & society.** *Nanomedicine (Lond)* 2015, **10**(21):3199-3202. doi:10.2217/nnm.15.153.

Jensen C, Forlini C, Partridge B, Hall W: **Australian university students’ coping strategies and use of pharmaceutical stimulants as cognitive enhancers.** *Front Psychol* 2016, **7**:277. doi:10.3389/fpsyg.2016.00277.

Jones DG: **Enhancement: are ethicists excessively influenced by baseless speculations?** Med Humanit 2006, **32**(2):77-81. doi:10.1136/jmh.2005.000234.

Kamm FM: **Is there a problem with enhancement?** *Am J Bioeth* 2005, **5**(3):5-14. doi:10.1080/15265160590945101.

Kraemer F: **Me, myself and my brain implant: deep brain stimulation raises questions of personal authenticity and alienation.** *Neuroethics* 2013, **6**:483-497. doi:10.1007/s12152-011-9115-7

Krstić P: **The better human, the better than human: limits of enhancement**. *Filozofija i Društvo* 2012, **23**(2):124-144. doi:10.2298/FID1202124K.

Kuiken T: **Nanomedicine and ethics: is there anything new or unique?** *Wiley Interdiscip Rev Nanomed Nanobiotechnol* 2011, **3**(2):111-118. doi:10.1002/wnan.90.

Lamkin M: **Regulating identity: medical regulation as social control**. *BYU L Rev* 2016, **2016**(2):503-574.

Lane MA, Ingram DK, Roth GS: **The serious search for an anti-aging pill**. *Sci Am* 2002, **287** (2):36-41. doi:10.1038/scientificamerican0802-36.

Larrieu P: **Les enjeux éthiques de la neuroamélioration [ethical issues of the neuroimprovement]**. *Médecine & Droit* 2014, **126**:61-65. doi:10.1016/j.meddro.2014.03.003.

Larriviere D, Williams MA: **Neuroenhancement: wisdom of the masses or “false phronesis”?** *Clin Pharmacol Ther* 2010, **88**(4):459-461. doi:10.1038/clpt.2010.140.

Levy N: **There may be costs to failing to enhance, as well as to enhancing**. *Am J Bioeth* 2013, **13**(7):38-39. doi:10.1080/15265161.2013.796222.

Liakoni E et al.: **The use of prescription drugs, recreational drugs, and "soft enhancers" for cognitive enhancement among Swiss secondary school students.** *PLoS One* 2015, **10**(10):e0141289. doi:10.1371/journal.pone.0141289.

Lucas MS: **Baby steps to superintelligence: neuroprosthetics and children.** *J Evol Technol* 2012, **22**(1):132-145.

Lucke J. & Patridge B: **Towards a smart population: a public health framework for cognitive enhancement.** *Neuroethics* 2013, **6**: 419. doi:10.1007/s12152-012-9167-3.

MacDonald C, Poirier N: **Pediatric neuroenhancement: full steam ahead, in a leaky boat?** *AJOB Neurosci* 2010, **1**(1):33-35. doi:10.1080/21507740903504517.

Maier LJ, Haug S, Schaub MP: **Author's response on Arria (2016): intention matters-using the terminology 'pharmacological neuroenhancement' as a behavioural definition based on the assumed functionality.** *Addiction* 2016, **111**(5):938-939. doi:10.1111/add.13331.

Maier LJ et al.: **Pharmacological cognitive enhancement in healthy individuals: a compensation for cognitive deficits or a question of personality?** *PLoS One* 2015, **10** (6):e0129805. doi:10.1371/journal.pone.0129805.

Maier LJ, Haug S, Schaub MP: **Prevalence of and motives for pharmacological neuroenhancement in Switzerland--results from a national Internet panel.** *Addiction* 2016, **111**(2):280-295. doi:10.1111/add.13059.

Maier LJ et al.: **Swiss university students’ attitudes toward pharmacological cognitive enhancement.** *PLoS One* 2015, **10**(12):e0144402. doi:10.1371/journal.pone.0144402.

Maier LJ, Liechti ME, Herzig F, Schaub MP: **To dope or not to dope: neuroenhancement with prescription drugs and drugs of abuse among Swiss university students.** *PLoS One* 2013, **8** (11):e77967. doi:10.1371/journal.pone.0077967.

Maslen H, Earp BD, Kadosh RC, Savulescu J: **Brain stimulation for treatment and enhancement in children: an ethical analysis**. *Front Hum Neurosci* 2014, **8**:1-5. doi:10.3389/fnhum.2014.00953.

Maslen H et al.: **The regulation of cognitive enhancement devices: refining Maslen et al.’s model**. *J Law Biosci* 2015, **2**(3):754-767. doi:10.1093/jlb/lsv029.

Mauron A, Hurst S: **From Ritalin to malignant teaching -- the fuzzy borders of neuroenhancement**. *AJOB Neurosci* 2010, **1**(1):31-33. doi:10.1080/21507740903504384.

McCrickerd J: **Sudden discontinuation and the subjective character of experience: a reason to resist psychotropic neuroenhancements**. *AJOB Neurosci* 2010, **1**(1):23-25. doi:10.1080/21507740903504459.

Mendelsohn D, Lipsman N, Bernstein M: **Neurosurgeons’ perspectives on psychosurgery and neuroenhancement: a qualitative study at one center.** *J Neurosurg* 2010, **113**(6): 1212-1218. doi:10.3171/2010.5.JNS091896.

Mohamed AD, Sahakian BJ: **The ethics of elective psychopharmacology**. *Int J Neuropsychopharmacol* 2012, **15**(4):559-571. doi:10.1017/S146114571100037X.

Mohamed AD: **Neuroethical issues in pharmacological cognitive enhancement.** *Wiley Interdiscip Rev Cogn Sci* 2014, **5**(5):533-549. doi:10.1002/wcs.1306.

Moutaud B: **Neuromodulation technologies and the regulation of forms of life: exploring, treating, enhancing**. *Med Anthropol* 2016, **35**(1):90-103. doi:10.1080/01459740.2015.1055355.

Normann C, Boldt J, Maio G, Berger M: **[Options, limits and ethics of pharmacological neuroenhancement].** *Nervenarzt* 2010, **81**(1):66-74. doi:10.1007/s00115-009-2858-2.

Ori L: **Should children have equal access to neuroenhancements?** *AJOB Neurosci* 2010, **1**(1):21-23. doi:10.1080/21507740903504442.

Ott R: **Neuroenhancement - perspectives of Swiss psychiatrists and general practitioners.** *Swiss Med Wkly* 2012, **142:**w13707. doi:10.4414/smw.2012.13707.

Outram SM, Racine E: **Examining reports and policies on cognitive enhancement: approaches, rationale, and recommendations.** *Account Res* 2011, **18**(5):323-341. doi:10.1080/08989621.2011.606734.

Outram SM: **Negotiating an inevitable future?** *AJOB Neurosci* 2010, **1**(1):29-31. doi:10.1080/21507740903504426.

Outram SM: **The use of methylphenidate among students: the future of enhancement?** *J Med Ethics* 2010, **36**(4):198-202. doi:10.1136/jme.2009.034421.

Parens E: **Authenticity and ambivalence: toward understanding the enhancement debate**. *Hastings Cent Rep* 2005, **35**(3):34-41. doi:10.1353/hcr.2005.0067.

Parens E: **On good and bad forms of medicalization**. *Bioethics* 2013, **27**(1):28-35. doi:10.1111/j.1467-8519.2011.01885.x.

Partridge BJ et al.: **Smart drugs “as common as coffee”: media hype about neuroenhancement.** *PLoS One* 2011, **6**(11):e28416. doi:10.1371/journal.pone.0028416.

Persson I, Savulescu J: **The perils of cognitive enhancement and the urgent imperative to enhance the moral character of humanity.** *J Appl Philos* 2008, **25**(3):162-177. doi:10.1111/j.1468-5930.2008.00410.x.

Penrose B: **Sandel on enhancement : a response to Van Niekerk**.

*S Afr J Philos* 2016, **35**(2):145-163. doi:10.1080/02580136.2016.1162038.

Pickersgill M, Hogle L: **Enhancement, ethics and society: towards an empirical research agenda for the medical humanities and social sciences.** *Med Humanit* 2015, **41**(2):136-142. doi:10.1136/medhum-2015-010718.

Ponnet K et al.: **Determinants of physicians’ prescribing behaviour of methylphenidate for cognitive enhancement.** *Psychol Health Med* 2014, **19**(3):286-295. doi:10.1080/13548506.2013.802361.

Racine E, Forlini C: **Responding to requests from adult patients for neuroenhancements: guidance of the Ethics, Law and Humanities Committee**. *Neurology* 2010, **74**(19):1555-1556. doi:10.1212/WNL.0b013e3181d8a54a.

Racine E et al.: **The value and pitfalls of speculation about science and technology in bioethics: the case of cognitive enhancement.** *Med Health Care Philos* 2014, **17**(3):325-337. doi:10.1007/s11019-013-9539-4.

Ragan CI, Bard I, Singh I, Independent Scientific Committee on Drugs: **What should we do about student use of cognitive enhancers? an analysis of current evidence.** *Neuropharmacology* 2013, **64**:588-595. doi:10.1016/j.neuropharm.2012.06.016.

Ray KS: **Not just "study drugs" for the rich: stimulants as moral tools for creating opportunities for socially disadvantaged students**. *Am J Bioeth* 2016, **16**(6):29-38. doi:10.1080/15265161.2016.1170231.

Rodríguez B: **Sobre la relevancia moral de la distinción mejora-tratamiento [on the moral relevance of the enhancement treatment distinction]**. *Dilemata* 2012, **10**:307-328.

Roduit JA, Baumann H, Heilinger JC: **Human enhancement and perfection.** *J Med Ethics* 2013, **39**(10):647-650. doi:10.1136/medethics-2012-100920.

Roduit JA, Heilinger JC, Baumann H: **Ideas of perfection and the ethics of human enhancement.** *Bioethics* 2015, **29**(9):622-630. doi:10.1111/bioe.12192.

Sahakian BJ, Morein-Zamir S: **Neuroethical issues in cognitive enhancement**. *J Psychopharmacol* 2011, **25**(2):197-204. doi:10.1177/0269881109106926.

Sandberg A, Bostrom N: **Converging cognitive enhancements**. *Ann N Y Acad Sci* 2006, **1093**:201-227. doi:10.1196/annals.1382.015.

Sandberg A: **Enhancement policy and the value of information**. *Am J Bioeth* 2013, **13**(7):34-35. doi:10.1080/15265161.2013.795826.

Sattler S, Schunck R: **Associations between the big five personality traits and the non-medical use of prescription drugs for cognitive enhancement.** *Front Psychol* 2016, **6**: 1971. doi:10.3389/fpsyg.2015.01971.

Sattler S, Mehlkop G, Graeff P, Sauer C: **Evaluating the drivers of and obstacles to the willingness to use cognitive enhancement drugs: the influence of drug characteristics, social environment, and personal characteristics.** *Subst Abuse Treat Prev Policy* 2014, **9**:8. doi: 10.1186/1747-597X-9-8.

Sattler S, Forlini C, Racine E, Sauer C: **Impact of contextual factors and substance characteristics on perspectives toward cognitive enhancement.** *PLoS One* 2013, **8**(8):e71452. doi:10.1371/journal.pone.0071452.

Savulescu J: **A liberal consequentialist approach to regulation of cognitive enhancers**. *Am J Bioeth* 2013, **13**(7):53-55. doi:10.1080/15265161.2013.796220.

Schelle KJ, Faulmüller N, Caviola L, Hewstone M: **Attitudes toward pharmacological cognitive enhancement-a review.** *Front Syst Neurosci* 2014, **8**:53. doi:10.3389/fnsys.2014.00053.

Schwartz PH: **Defending the distinction between treatment and enhancement**. *Am J Bioeth* 2005, **5**(3):17-19. doi:10.1080/15265160591002755.

Selgelid MJ: **An argument against arguments for enhancement**. *Stud Ethics Law Technol* 2008, **1**(1):12. doi:10.2202/1941-6008.1008.

Singh I, Kelleher KJ: **Neuroenhancement in young people: proposal for research, policy, and clinical management**. *AJOB Neurosci* 2010, **1**(1):3-16. doi:10.1080/2150774090350859.

Synofzik M: **Kognition à la carte? - der wunsch nach kognitionsverbessernden psychopharmaka in der medizin [cognition on demand? - the wish for cognition-enhancing drugs in medicine]**. *Ethik Med* 2006, **18**(1):37-50. doi:10.1007/s00481-006-0412-3.

Synofzik M, Schlaepfer TE: **Stimulating personality: ethical criteria for deep brain stimulation in psychiatric patients and for enhancement purposes**. *Biotechnol J* 2008, **3**(12):1511-1520. doi:10.1002/biot.200800187.

Sparrow R: **Enhancement and obsolescence: avoiding an “enhanced rat race”.** *Kennedy Inst Ethics J* 2015, **25**(3):231-260. doi:10.1353/ken.2015.0015.

Trancik EK: **Enhancement versus therapy in Catholic neuroethics**. *Natl Cathol Bioeth Q* 2015, **15**(1):63-72. doi:10.5840/ncbq20151517.

Vargo EJ, Petróczi A: **“It was me on a good day”: exploring the smart drug use phenomenon in England.** *Front Psychol* 2016, **7**:779. doi:10.3389/fpsyg.2016.00779.

Wade L, Forlini C, Racine, E: **Generating genius: how an Alzheimer's drug became considered a 'cognitive enhancer' for healthy individuals.** *BMC Med Ethics* 2014, **15**:37. doi:10.1186/1472-6939-15-37.

Walcher-Andris E: **Ethische aspekte des pharmakologischen „cognition enhancement“ am beispiel des gebrauchs von psychostimulanzien durch kinder und jugendliche [ethical aspects of pharmacological "cognition enhancement" using the example of the use of psychostimulants by children and adolescents]**. *Ethik Med* 2006, **18**(1):27-36. doi:10.1007/s00481-006-0411-4.

Wolff W, Baumgarten F, Brand R: **Reduced self-control leads to disregard of an unfamiliar behavioral option: an experimental approach to the study of neuroenhancement.** *Subst Abuse Treat Prev Policy* 2013, **8**:41. doi:10.1186/1747-597X-8-41.

Wolff W, Sandouqa Y, Brand R: **Using the simple sample count to estimate the frequency of prescription drug neuroenhancement in a sample of Jordan employees.** *Int J Drug Policy* 2016, **31**:51-55. doi:10.1016/j.drugpo.2015.12.014.

Wolpe PR: **Treatment, enhancement, and the ethics of neurotherapeutics**. *Brain Cogn* 2002, **50**(3):387-395. doi:10.1016/S0278-2626(02)00534-1.

Zelli A, Lucidi F, Mallia L: **The complexity of neuroenhancement and the adoption of a social cognitive perspective.** *Front Psychol* 2015, **6**:1880. doi:10.3389/fpsyg.2015.01880.

Zwart H: **Limitless as a neuro-pharmaceutical experiment and as a Daseinsanalyse: on the use of fiction in preparatory debates on cognitive enhancement.** *Med Health Care Philos* 2014, **17**(1):29-38. doi:10.1007/s11019-013-9481-5.

**Books:**

Agar N: *Humanity’s End: Why We Should Reject Radical Enhancement*. Cambridge, Mass: MIT Press; 2010.

Agar N: *Liberal Eugenics: In Defence of Human Enhancement*. Malden, MA: Blackwell Publishing; 2005.

Agar N: *Truly Human Enhancement: A Philosophical Defense of Limits*. Cambridge, Mass: MIT Press; 2014.

Baron J: *Against Bioethics.* Cambridge, MA: MIT Press; 2006.

Blank RH: *Cognitive Enhancement: Social and Public Policy Issues*. New York: Palgrave Pivot; 2015.

Basl J, Sandler RL, eds.: *Designer Biology: The Ethics of Intensively Engineering Biological and Ecological Systems*. Lanham: Lexington Books; 2013.

Bateman S et al. eds.: *Inquiring Into Human Enhancement: Interdisciplinary and International Perspectives*. New York: Palgrave Macmillan; 2015.

Buchanan AE: *Beyond Humanity? The Ethics of Biomedical Enhancement*. Oxford: Oxford University Press; 2011.

Cabrera LY: *Rethinking Human Enhancement: Social Enhancement and Emergent Technologies*. New York: Palgrave Macmillan; 2015.

Cassell E: *The Nature of Suffering and the Goals of Medicine.* Oxford: Oxford University Press; 2004.

Coeckelbergh M: *Human Being @ Risk: Enhancement, Technology, and the Evaluation of Vulnerability Transformations*. Dordrecht: Springer; 2013.

Dickenson D: *Me Medicine vs. We Medicine: Reclaiming Biotechnology for the Common Good*. New York: Columbia University Press; 2013.

Eilers M, Gruber K, Rehmann-Sutter C, eds: *The Human Enhancement Debate and Disability: New Bodies for a Better Life*. New York: Palgrave Macmillan; 2014.

Engel AK, Friston KJ, Kragic D: *The Pragmatic Turn: Toward Action-Oriented Views in Cognitive Science*. Cambridge, Mass.: MIT Press; 2015.

Flanagan O: *The Problem of the Soul: Two Visions of Mind and How to Reconcile Them.* New York, NY: Basic Books; 2002.

Fröding B, Osika W: *Neuroenhancement: How Mental Training and Meditation Can Promote Epistemic Virtue*. Cham, Switzerland: Springer International Publishing; 2015.

Glannon W: *Bioethics and the Brain.* Oxford: Oxford University Press; 2006.

Gordijn B, Chadwick R, eds.: *Medical Enhancement and Posthumanity*. Dordrecht: Springer; 2008.

Hauskeller M: *Better Humans? Understanding the Enhancement Project*. Durham, UK: Acumen; 2013.

Hildt E, Franke AG: *Cognitive Enhancement: An Interdisciplinary Perspective*. Dordrecht: Springer; 2013.

Jotterand F, Dubljević V, eds.: *Cognitive Enhancement: Ethical and Policy Implications in International Perspectives*. New York: Oxford University Press; 2016.

Kass L: *Beyond Therapy: Biotechnology and the Pursuit of Happiness.* Washington, D.C.: President's Council on Bioethics; 2003.

Mehlman MJ: *The Price of Perfection: Individualism and Society in the Era of Biomedical Enhancement*. Baltimore: Johns Hopkins University Press; 2009.

Naam R: *More Than Human: Embracing the Promise of Biological Enhancement*. New York: Broadway Books; 2005.

Parens E: *Shaping Our Selves: On Technology, Flourishing, and a Habit of Thinking*. Oxford: Oxford University Press; 2015.

Roduit JAR: *The Case for Perfection: Ethics in the Age of Human Enhancement*. New York: Peter Lang; 2016.

Rothman SM: *The Pursuit of Perfection: The Promise and Perils of Medical Enhancement*. New York: Vintage Books; 2004.

Sadler J: *Values and Psychiatric Diagnosis.* Oxford: Oxford University Press; 2005.

Savulescu J, Bostrom N, eds.: *Human Enhancement*. Oxford: Oxford University Press; 2009.

Schöne-Seifert B et al.: *Neuro-Enhancement : Ethik vor neuen Herausforderungen*. Paderborn: Mentis; 2009.

Schütz R, Hildt E, Hampel J: *Neuroenhancement Interdisziplinäre Perspektiven auf eine Kontroverse [Neuroenhancement: Interdisciplinary Perspectives on a Controversy].* Bielefeld: transcript Verlag; 2016.

Sisti D, Caplan A, McCartney J: *Health, Disease, and Illness: Concepts in Medicine.* Washington, D.C.: Georgetown University Press; 2004.

Vincent JD: The Custom-Made Brain: Cerebral Plasticity, Regeneration, and Enhancement. New York: Columbia University Press; 2014.

Wiseman H: *The Myth of the Moral Brain: The Limits of Moral Enhancement*. Cambridge, Mass: MIT Press; 2016.

Wolpe P: *Introductory Remarks to the Panel.* First Meeting of Neuroethics Society. Washington, D.C.; 2008.

**Book Chapters:**

Abney K, Lin P, Mehlman M: **Military neuroenhancement and risk assessment**. In *Neurotechnology in National Security and Defense: Practical Considerations, Neuroethical Concerns*. Edited by James Giordano. Boca Raton: CRC Press; 2015:239-248.

Blank RH: **Ethical and social context of cognitive enhancement**. In his *Cognitive Enhancement: Social and Public Policy Issues*. New York: Palgrave Pivot; 2015:42-70.

Cabrera LY: **How does enhancing cognition affect human values? how does this translate into social responsibility?** In *Ethical Issues in Behavioral Neuroscience*. Edited by Grace Lee, Judith Illes, Frauke Ohl. Heidelberg: Springer; 2015:223-241.

Caplan AL: **An unnatural process : why is it not inherently wrong to seek a cure for aging?** In *Fountain of Youth: Cultural, Scientific, and Ethical Perspectives on a Biomedical Goal*. Edited by Stephen G. Post, Robert H. Binstock. Oxford: Oxford University Press; 2004:271-285.

Caplan AL, McHugh PR: **Shall we enhance? a debate.** In *Defining Right and Wrong in Brain Science*. Edited by Walter Glannon. New York: Dana Press; 2007:271-288.

Chancellor B, Chatterjee A: **Brain training.** In *Neuroethics in Practice.* Edited by Anjan Chatterjee, Martha J. Farah. New York, NY: Oxford University Press; 2013:57-68.

Chatterjee A: **Brain enhancement in healthy adults.** In *Neuroethics in Practice.* Edited by Anjan Chatterjee, Martha J. Farah. New York, NY: Oxford University Press; 2013:3-15.

Conrad P, Horwitz A: **Marketing of neuropsychiatric illness and enhancement.** In *Neuroethics in Practice.* Edited by Anjan Chatterjee, Martha J. Farah. New York, NY: Oxford University Press; 2013:46-56.

Dominey PF, Prescott TJ, Bohg J, Schwartz A: **Implications of action-oriented paradigm shifts in cognitive science**. In *The Pragmatic Turn: Toward Action-Oriented Views in Cognitive Science*. Edited by Andreas K. Engel, Karl Friston, Danica Kragic. Cambridge, Mass.: MIT Press; 2015:333-356.

Farah MJ et al.: **Neurocognitive enhancement: what can we do and what should we do?** In *Defining Right and Wrong in Brain Science*. Edited by Walter Glannon. New York: Dana Press; 2007:289-301.

Gini A, Giordano J: **The human condition and strivings to flourish**. In *Scientific and Philosophical Perspectives in Neuroethics*. Edited by James J. Giordano, Bert Gordijn. Cambridge: Cambridge University Press; 2010:343-354.

Glannon W: **Cognitive enhancement.** In his *Brain, Body, and Mind.* New York, NY: Oxford University Press, Inc.; 2011:115-145.

Klitzman R: **Clinicians, patients, and the brain**. In *Neuroethics: Defining the Issues in Theory, Practice, and Policy*. Edited by Judith Illes. Oxford: Oxford University Press; 2006:229-244.

Parens E: **How far will the treatment/enhancement distinction get us as we grapple with new ways to shape ourselves?** In *Neuroethics: Mapping the Field*. Edited by Steven J. Marcus. New York: Dana Press; 2002:152-158.

Presidential Commission for the Study of Bioethical Issues. **Cognitive enhancement and beyond**. In their *Gray Matters: Topics at the Intersection of Neuroscience, Ethics, and Society (Vol. 2)*. Washington, DC: Presidential Commission for the Study of Bioethical Issues; 2015:27-52.

Racine E: **Enhancement of performance with neuropharmaceuticals: pragmatism and the culture wars.** In his *Pragmatic Neuroethics: Improving Treatment and Understanding of the Mind-Brain.* Cambridge, MA: MIT Press; 2010:121-138.

Racine D, DuRousseau D, Illes J: **Ethical issues in performance-enhancing technologies: from bench to headline**. In *Neurotechnology: Premises, Potential, and Problems*. Edited by James Giordano. Boca Raton: CRC Press; 2012:175-198.

Russo MB, Stetz MC, Stetz TA: **Ethical considerations: cogniceuticals in the military.** In *Neuroethics in Practice.* Edited by Anjan Chatterjee, Martha J. Farah. New York, NY: Oxford University Press; 2013:35-45.

Siipi H: **Is neuroenhancement unnatural, and does it morally matter?** In *Neurotechnology: Premises, Potential, and Problems*. Edited by James Giordano. Boca Raton: CRC Press; 2012:199-212.

Singh I, Kelleher K: **The case for clinical management of neuroenhancement in young people.** In *Neuroethics in Practice.* Edited by Anjan Chatterjee, Martha J. Farah. New York, NY: Oxford University Press; 2013:16-34.

Terbeck S: **Neuroethics of social enhancement**. In her *The Social Neuroscience of Intergroup Relations: Prejudice, Can We Cure It?* New York: Springer; 2016:69-83.

Viirre E, Baylis F, Downie J: **Promises and perils of cognitive performance tools: a dialogue**. In *Neurotechnology: Premises, Potential, and Problems*. Edited by James Giordano. Boca Raton: CRC Press; 2012:125-141.

### Ethical issues in neurology (including neuro-rehabilitation)

**Articles:**

Al-Delaimy WK: **Ethical concepts and future challenges of neuroimaging: an Islamic perspective**. *Sci Eng Ethics* 2012, **18**(3):509-518. doi:10.1007/s11948-012-9386-3

Andrews K: **Medical decisionmaking in the vegetative state: withdrawal of nutrition and hydration**. *NeuroRehabilitation* 2004, **19**(4):299-304.

Annas GJ: **“Culture of life” politics at the bedside - the case of Terri Schiavo**. *N Engl J Med* 2005, **352(16)**:1710-1715. doi:10.1056/NEJMlim050643.

Aricò I et al.: **Could combined sleep and pain evaluation be useful in the diagnosis of disorders of consciousness (DOC)? preliminary findings**. *Brain Inj* 2016, **30**(2):159-163. doi: 10.3109/02699052.2015.1089595.

Baars BJ, Ramsøy TZ, Laureys S: **Brain, conscious experience and the observing self.** *Trends Neurosci* 2003, **26**(12):671-675. doi:10.1016/j.tins.2003.09.015.

Bardin JC et al.: **Dissociations between behavioural and functional magnetic resonance imaging-based evaluations of cognitive function after brain injury**. *Brain* 2011, **134**(Pt 3): 769-782. doi: 10.1093/brain/awr005.

Bender A et al.: **Persistent vegetative state and minimally conscious state: a systematic review and meta-analysis of diagnostic procedures.** *Dtsch Arztebl Int* 2015, 112(14):235-242. doi:10.3238/arztebl.2015.0235.

Bernat JL: **The biophilosophical basis of whole-brain death**. *Soc Philos Policy* 2002, **19**(2): 324-342. doi:10.1017/S0265052502192132.

Brogan ME, Provencio JJ: **Spectrum of catastrophic brain injury: coma and related disorders of consciousness.** *J Crit Care* 2014, **29**(4):679-682. doi:10.1016/j.jcrc.2014.04.014.

Brosnan C, Cribb A, Wainwright SP, Williams C: **Neuroscientists' everyday experiences of ethics: the interplay of regulatory, professional, personal and tangible ethical spheres**. *Sociol Health Illn* 2013, **35**(8):1133-1148. doi:10.1111/1467-9566.12026.

Calabrò RS et al.: **End-of-life decisions in chronic disorders of consciousness: sacrality and dignity as factors**. *Neuroethics* 2016, **9**(1):85-102. doi:10.1007/s12152-016-9257-8.

Clausen J: **Moving minds: ethical aspects of neural motor prostheses**. *Biotechnol J* 2008, **3**(12):1493-1501. doi:10.1002/biot.200800244.

Comte A et al.: **On the difficulty to communicate with fMRI-based protocols used to identify covert awareness**. *Neuroscience* 2015, **300**:448-459. doi:10.1016/j.neuroscience.2015.05.059.

Conrad G, Sinha P: **Scintigraphy as a confirmatory test of brain death**. *Semin Nucl Med* 2003, **33**(4):312-323. doi:10.1016/S0001-2998(03)00034-5.

Cranford R: **Facts, lies, and videotapes: the permanent vegetative state and the sad case of Terri Schiavo**. *J Law Med Ethics* 2005, **33**(2):363-371. doi:10.1111/j.1748-720X.2005.tb00501.x.

Crozier S: **Neuroéthique: questions éthiques en neurosciences [neuroethics: ethical issues in neurosciences]**. *Rev Prat* 2013, **63**(5):666-669.

Deeb W et al.: **Proceedings of the Fourth Annual Deep Brain Stimulation Think Tank: a review of emerging issues and technologies**. *Front Integr Neurosci* 2016, **10**:38. doi:10.3389/fnint.2016.00038.

Demetriades AK, Demetriades CK, Watts C, Ashkan K: **Brain-machine interface: the challenge of neuroethics**. *Surgeon* 2010, **8**(5):267-269. doi:10.1016/j.surge.2010.05.006.

Farah MJ: **An ethics toolbox for neurotechnology**. *Neuron* 2015, **86**(1):34-37. doi:10.1016/j.neuron.2015.03.038.

Farah MJ: **Neuroethics and the problem of other minds: implications of neuroscience evidence for the moral status of brain-damaged patients and nonhuman animals**. *Neuroethics* 2008, **1**(1):9-18. doi:10.1007/s12152-008-9006-8.

Farah MJ, Heberlein AS: **Personhood and neuroscience: naturalizing or nihiliating?** *Am J Bioeth* 2007, **7**(1):37-48. doi:10.1080/15265160601064199.

Farisco M, Petrini C: **Misdiagnosis as an ethical and scientific challenge.** *Ann Ist Super Sanita* 2014, **50**(3):229-233. doi:10.4415/ANN_14_03_05.

Figueroa G: **Neuroética: reflexiones sobre los principios de la moral latentes en la medicina [neuroethics: reflections on the latent principles of morals in medicine]**. *Rev Med Chil* 2012, **140**(8):1078-1084. doi:10.4067/S0034-98872012000800018.

Fins JJ: **Clinical pragmatism and the care of brain damaged patients: toward a palliative neuroethics for disorders of consciousness.** Prog Brain Res 2005, 150:565-582. doi:10.1016/S0079-6123(05)50040-2.

Fins JJ: **Lessons from the injured brain: a bioethicist in the vineyards of neuroscience**. *Camb Q Healthc Ethics* 2009, **18**(1):7-13. doi:10.1017/S0963180108090038.

Fins JJ et al. **Neuroimaging and disorders of consciousness: envisioning an ethical research agenda**. *Am J Bioeth* 2008, **8**(9):3-12. doi:10.1080/15265160802318113.

Fins JJ: **Nanotechnology, neuromodulation & the immune response: discourse, materiality & ethics.** *Biomed Microdevices* 2015, **17**(2):28. doi:10.1007/s10544-015-9934-0.

Fins JJ, Schiff ND: **Shades of gray: new insights into the vegetative state**. *Hastings Cent Rep* 2006, **36**(6):8. doi:10.1353/hcr.2006.0094.

Ford PJ, Deshpande A: **The ethics of surgically invasive neuroscience research**. *Handb Clin Neurol* 2013, **118**:315-321. doi:10.1016/B978-0-444-53501-6.00026-3.

Gabriel D et al.: **Replicability and impact of statistics in the detection of neural responses of consciousness.** *Brain* 2016**, 139**(Pt 6):e30. doi:10.1093/brain/aww065.

Gassen HG: **Why neuroethics?** *Biotechnol J* 2008, **3**(12):1463-1465. doi:10.1002/biot.200890109.

Giacino JT et al.: **The minimally conscious state: definition and diagnostic criteria.** *Neurology* 2002, **58**(3):349-353. doi:10.1212/WNL.58.3.349.

Giordano J: **Conditions for consent to the use of neurotechnology: a preparatory neuroethical approach to risk assessment and reduction**. *AJOB Neurosci* 2015, **6**(4):12-49. doi:10.1080/21507740.2015.1094557.

Giordano J, Akhouri R, McBride DK: **Implantable nano-neurotechnological devices: consideration of ethical, legal and social issues and implications.** *J Long Term Eff Med Implants* 2009, **19**(1)**:**83-93. doi:10.1615/JLongTermEffMedImplants.v19.i1.80.

Giordano J: **A preparatory neuroethical approach to assessing developments in neurotechnology**. *AMA J Ethics* 2015, **17**(1):56-61. doi:10.1001/virtualmentor.2015.17.01.msoc1-1501.

Giordano J. **Toward an operational neuroethical risk analysis and mitigation paradigm for emerging neuroscience and technology (neuroS/T)**. *Exp Neurol* 2016, **287**(4):492-495. doi:10.1016/j.expneurol.2016.07.016.

Giordano J: **The value of patient benefit: consideration of framing contingencies to guide the ethical use of DBS--a case analysis**. *Camb Q Healthc Ethics* 2016, **25**(4):755-758. doi:10.1017/S0963180116000530.

Gostin LO: **Ethics, the constitution, and the dying process: the case of Theresa Marie Schiavo.** *JAMA* 2005, **293**(19):2403-2407. doi:10.1001/jama.293.19.2403.

Green RM: **Neural technologies: the ethics of intimate access to the mind**. *Hastings Cent Rep* 2015, **45**(6):36-37. doi:10.1002/hast.516.

Greenspan RJ: **Biological indeterminacy**. *Sci Eng Ethics* 2012, **18**(3):447-452. doi:10.1007/s11948-012-9379-2.

Harris J, Lawrence DR: **Hot baths and cold minds: neuroscience, mind reading, and mind misreading**. *Camb Q Healthc Ethics* 2015, **24**(2):123-134. doi:10.1017/S0963180114000425.

Haubenberger D, Clifford DB: **Clinical trials in neurovirology: successes, challenges, and pitfalls**. *Neurotherapeutics* 2016, **13**(3):571-581. doi:10.1007/s13311-016-0440-8.

Illes J: **Empirical neuroethics: can brain imaging visualize human thought? why is neuroethics interested in such a possibility**. *EMBO Rep* 2007, **8**:S57-S60. doi:10.1038/sj.embor.7401007.

Iosa M, Morone G, Cherubini A, Paolucci S: **The three laws of neurorobotics: a review on what neurorehabilitation robots should do for patients and clinicians**. *J Med Biol Eng* 2016, **36**:1-11. doi:10.1007/s40846-016-0115-2.

Jennett B: **The assessment and rehabilitation of vegetative and minimally conscious patients: definitions, diagnosis, prevalence and ethics.** *Neuropsychological Rehabilitation* 2005, **15**(3-4):163-165. doi:10.1080/09602010443000632.

Jox RJ, Schöne-Seifert B, Brukamp K: **Aktuelle kontroversen in der neuroethik [current controversies in neuroethics]**. *Nervenarzt* 2013, **84**(10):1163-1164. doi:10.1007/s00115-013-3731-x.

Jox RJ: **Beste interessen im "vegetativen zustand" [best interests in the 'vegetative state']**. *Fortschr Neurol Psychiatr* 2011, **79**(10):576-581. doi:10.1055/s-0031-1281736.

Jox RJ et al.: **Diagnosis and decision making for patients with disorders of consciousness: a survey among family members**. *Arch Phys Med Rehabil* 2015, **96**(2):323-330. doi: 10.1016/j.apmr.2014.09.030.

Jox RJ, Bernat JL, Laureys, Racine E: **Disorders of consciousness: responding to requests for novel diagnostic and therapeutic interventions.** *Lancet Neurol* 2012, **11**(8):732-738. doi: 10.1016/S1474-4422(12)70154-0.

Kawashima K: **終末期のケアと神経科学における最善の生活を考える [thinking about the best life in end-of-life care and neuroethics]**. *Rinsho Shinkeigaku* 2008, **48**(11):955-957.

Kontsevenko AS: **Биoэтичecкиe пpoблeмы в нeйpoбиoлoгии [bioethical problems in neurosciences]**. *Zh Nevrol Psikhiatr Im S S Korsakova* 2011, **111**(4):67-70.

Krug H: **Neuroethik in der klinischen praxis [Neuroethics in clinical practice]**. *Nervenarzt* 2009, **80**(8):941-947. doi:10.1007/s00115-009-2683-7.

Kuehlmeyer K et al.: **Physicians' attitudes toward medical and ethical challenges for patients in the vegetative state: comparing Canadian and German perspectives in a vignette survey.** *BMC Neurol* 2014, **14:**119. doi:10.1186/1471-2377-14-119.

Kushner T, Giordano J: **Clinical neuroethics – from bench to bedside…and beyond**. *Camb Q Healthc Ethics* 2016, **25**(4):570-572. doi:10.1017/S096318011600030X.

Lanfermann H, Schober O: **Imaging of irreversible loss of brain function**. *Röfo* 2016, **188**(1):23-26. doi:10.1055/s-0041-108202.

Latronico N, Manenti O, Baini L, Rasulo FA: **Quality of reporting on the vegetative state in Italian newspapers: the case of Eluana Englaro**. *PLoS One* 2011, **6**(4):e18706. doi:10.1371/journal.pone.0018706.

Laureys S: **The neural correlate of (un)awareness: lessons from the vegetative states.** *Trends Cogn Sci* 2005, **9**(12):556-559. doi:10.1016/j.tics.2005.10.010.

Laureys S et al.: **Cerebral processing in the minimally conscious state**. *Neurology* 2004, **63**(5): 916-918. doi:10.1212/01.WNL.0000137421.30792.9B.

Lee G et al.: **Canadian perspectives on the clinical actionability of neuroimaging in disorders of consciousness**. *Can J Neurol Sci* 2015, **42**(2):96-105. doi:10.1017/cjn.2015.8.

Leshner AL: **Ethical issues in taking neuroscience research from bench to bedside**. *Cerebrum* 2004, **6**(4):66-72.

McMillan TM, Herbert CM: **Further recovery in a potential treatment withdrawal case 10 years after brain injury**. *Brain Inj* 2004, **18**(9):935-940. doi:10.1080/02699050410001675915.

Mikulis D, Weijer C, Young GB: **Illuminating awareness: implications of fMRI research in disorders of consciousness**. *Can J Neurol Sc*i 2015, **42**(4):211-212. doi:10.1017/cjn.2015.34.

Miller FG, Kaptchuk TJ: **Deception of subjects in neuroscience: an ethical analysis**. *J Neurosci* 2008, **28**(19):4841-4843. doi:10.1523/JNEUROSCI.1493-08.2008.

Moosa E: **Translating neuroethics: reflections from Muslim ethics: commentary on "Ethical concepts and future challenges of neuroimaging: an islamic perspective"**. *Sci Eng Ethics* 2012, **18**(3):519-528. doi:10.1007/s11948-012-9392-5.

Niebrój LT: **Neuroetyki: nowa jakość etyki lekarskiej? [Neuroethics: new quality of medical ethics?]** *Ann Acad Med Stetin* 2013, **59**(1):130-136.

Northoff G: **Methodische defizite in der neuroethik: brauchen wir theoretische neuroethik? [methodological deficits in neuroethics: do we need theoretical neuroethics?]** *Nervenarzt* 2013, **84**(10):1196-1202. doi:10.1007/s00115-013-3732-9.

Owen AM, Coleman MR: **Detecting awareness in the vegetative state.** *Ann N Y Acad Sci* 2008, **1129**:130-138. doi:10.1196/annals.1417.018.

Parker LS, Kienholz ML: **Disclosure issues in neuroscience research**. *Account Res* 2008, **15**(4):226-241. doi:10.1080/08989620802388697.

Peterson A et al.: **Risk, diagnostic error, and the clinical science of consciousness.** *Neuroimage Clin* 2015, **7**:588-597. doi:10.1016/j.nicl.2015.02.008.

Pierce R: **What a tangled web we weave: ethical and legal implications of deception in recruitment**. *Account Res* 2008, **15**(4):262-282. doi:10.1080/08989620802388713.

Pirruccello A: **Reductionism, brain imaging, and social identity: commentary on “biological indeterminacy”**. *Sci Eng Ethics* 2012, **18**(3):453-456. doi:10.1007/s11948-012-9372-9.

Pope John Paul II: **Address of Pope John Paul II to the participants in the International Congress on “life-sustaining treatments and vegetative state: scientific advances and ethical dilemmas.”** *NeuroRehabilitation* 2004, **19**(4):273-275.

Quill TE: **Terri Schiavo--a tragedy compounded**. *N Engl J Med* 2005, **352**(16):1630-1633. doi:10.1056/NEJMp058062.

Racine E et al.: **Proven or unproven? panel report on ethics in the translation of neuroscience**. *Can J Neurol Sci* 2012, **39**(2):247-250. doi:10.1017/S0317167100013330.

Rady MY, Verheijde JL: **Neuroscience and awareness in the dying human brain: implications for organ donation practices.** *J Crit Care* 2016, **34**:121-123. doi: 10.1016/j.jcrc.2016.04.016.

Robles del Olmo B, Garcia Collado D: [**Ethical challenges of the finding of covert awareness with neuroimaging in vegetative states.]** *Med Clin (Barc)* 2016, **146**(5):218-222. doi: 10.1016/j.medcli.2015.07.011.

Rossi PJ et al.: **Decision technologies in medical research and practice: Practical considerations, ethical implications and the need for dialectical evaluation**. *Ethics Biol Eng Med* 2013, **4**(2):91-102. doi:10.1615/EthicsBiologyEngMed.2013008091.

Rossi PJ, Giordano J, Walter BL, Okun MS: **Ethical considerations of broadcasting awake brain stimulation surgery: reigniting a debate**. *Brain Stimul* 2016, **9**(3):320-322. doi:10.1016/j.brs.2016.03.012.

Rossi PJ et al.: **Proceedings of the Third Annual Deep Brain Stimulation Think Tank: a review of emerging issues and technologies**. *Front Neurosci* 2016, **10**(119). doi:10.3389/fnins.2016.00119.

Rossi PJ, Okun M, Giordano J: **Translational imperatives in deep brain stimulation research: addressing neuroethical issues of consequences and continuity of clinical care.** *AJOB Neurosci* 2014, **5**(1): 46-48. doi:0.1080/21507740.2013.863248.

Saposnik G, Maurino J, Saizar R, Bueri JA: **Spontaneous and reflex movements in 107 patients with brain death**. *Am J Med* 2005, **118**(3):311-314. doi:10.1016/j.amjmed.2004.09.013.

Schiff ND et al.: **fMRI reveals large-scale network activation in minimally conscious patients.** *Neurology* 2005, **64**(3):514-523. doi:10.1212/01.WNL.0000150883.10285.44.

Schiff ND et al.: **Residual cerebral activity and behavioural fragments can remain in the persistently vegetative brain**. *Brain* 2002, **125**(Pt.6):1210-1234. doi:10.1093/brain/awf131.

Schorr B, Schlee W, Arndt M, Bender A: **Coherence in resting-state EEG as a predictor for the recovery from unresponsive wakefulness syndrome.** *J Neurol* 2016, **263**(5):937-953. doi: 10.1007/s00415-016-8084-5.

Smart CM, Giacino JT: **Exploring caregivers' knowledge of and receptivity toward novel diagnostic tests and treatments for persons with post-traumatic disorders of consciousness.** *NeuroRehabilitation* 2015, **37**(1):117-130. doi:10.3233/NRE-151244.

Trimper JB, Wolpe PR, Rommelfanger KS: **When “I” becomes “we”: ethical implications of emerging brain-to-brain interfacing technologies**. *Front Neuroeng* 2014, **7**:4. doi:10.3389/fneng.2014.00004.

Van McCrary S: **Transferring emerging neuroscience to the clinical ethics bedside**. *Am J Bioeth* 2009, **9**(9):21-23. doi:10.1080/15265160903098549.

Veatch RM: **Abandon the dead donor rule or change the definition of death?** *Kennedy Inst Ethics J* 2004, **14**(3):261-276. doi:10.1353/ken.2004.0035.

Venturelli AN, Branca I: **Evidencia y neurociencias cognitivas: el caso de la resonancia magnética funcional [evidence and cognitive neurosciences: the case of functional MRI]**. *Tópicos* 2016, **50**:177-207.

Wolpe PR: **Ethical and social challenges of brain-computer interfaces**. *Virtual Mentor* 2007, **9**(2):128-131. doi:10.1001/virtualmentor.2007.9.2.msoc1-0702.

**Books:**

Ackerman S: *Hard Science, Hard Choices: Facts, Ethics, and Policies Guiding Brain Science Today*. New York: Dana Press; 2006.

Bernat JL, Beresford HR: *Ethical and Legal Issues in Neurology*. Edinburgh: Elsevier; 2013.

Giordano JJ, Gordijn B: *Scientific and Philosophical Perspectives in Neuroethics*. Cambridge: Cambridge University Press; 2010.

Glannon W: *Defining Right and Wrong in Brain Science: Essential Readings in Neuroethics*. New York: Dana Press; 2007.

Jennett B: *The Vegetative State. Medical Facts, Ethical And Legal Dilemmas*. Cambridge: Cambridge University Press; 2002.

Jox RJ, International Neuroethics Workshop for Young Academics and Health Care Practitioners. *Vegetative State - A Paradigmatic Problem of Modern Society: Medical, Ethical, Legal and Social Perspectives on Chronic Disorders of Consciousness*. Wein: Lit-Verl; 2012.

Laureys S, Tononi G: *The Neurology of Consciousness: Cognitive Neuroscience and Neuropathology*. London: Academic; 2009.

Lavazza A: *Frontiers in Neuroethics: Conceptual and Empirical Advancements*. Newcastle upon Tyne: Cambridge Scholars Publishing; 2016.

Shevell MI: *Ethical Issues in Pediatric Neurology*. Philadelphia: Saunders; 2002.

Sinnott-Armstrong W: *Finding Consciousness: The Neuroscience, Ethics, and Law of Severe Brain Damage*. New York: Oxford University Press; 2016.

Williams MA, McGuire D, Rizzo M: *Practical Ethics in Clinical Neurology: A Case-based Learning Approach*. Philadelphia: Lippincott Williams & Wilkins; 2012.

**Book Chapters:**

Balaban CD: **Neurotechnology and operational medicine**. In *Neurotechnology in National Security and Defense: Practical Considerations, Neuroethical Concerns*. Edited by James Giordano. Boca Raton: CRC Press; 2015:65-78.

Bernat JL: **Consciousness and death: the whole-brain formulation of death**. In *Finding Consciousness: The Neuroscience, Ethics, and Law of Severe Brain Damage*. Edited by Walter Sinnott-Armstrong. New York: Oxford University Press; 2016:38-56.

Farah MJ: **Personhood, consciousness, and severe brain damage.** In *Neuroethics in Practice.* Edited by Anjan Chatterjee, Martha J. Farah. New York, NY: Oxford University Press; 2013: 175-188.

Fins JJ, Schiff ND: **Disorders of consciousness following severe brain injury.** In *Neuroethics in Practice.* Edited by Anjan Chatterjee, Martha J. Farah. New York, NY: Oxford University Press; 2013:162-174.

Glannon W: **Brain death.** In his *Bioethics and the Brain.* New York, NY: Oxford University Press; 2007:148-178.

Hawkins J: **What is good** ***for them*** **? Best interests and severe disorders of consciousness**. In *Finding Consciousness: The Neuroscience, Ethics, and Law of Severe Brain Damage*. Edited by Walter Sinnott-Armstrong. New York: Oxford University Press; 2016:180-206.

Laureys S: **Brain Death.** In *Neuroethics in Practice.* Edited by Anjan Chatterjee, Martha J. Farah. New York, NY: Oxford University Press; 2013:149-161.

Leshner AI: **Ethical issues in taking neuroscience research from bench to bedside**. In *Defining Right and Wrong in Brain Science*. Edited by Walter Glannon. New York: Dana Press; 2007:75-82.

Owen AM, Naci L: **Decoding thoughts in behaviorally nonresponsive patients**. In *Finding Consciousness: The Neuroscience, Ethics, and Law of Severe Brain Damage*. Edited by Walter Sinnott-Armstrong. New York: Oxford University Press; 2016:100-121.

Racine E: **Communication of prognosis in disorders of consciousness and severe brain injury: a closer look at paradoxical discourses in the clinical and public domains.** In his *Pragmatic Neuroethics.* Cambridge, MA: MIT Press; 2010:161-178.

Verheijde JL: **Evidence and ethics**. In *Evidence-based Neurology: Management of Neurological Disorders*. Edited by Bart M. Demaerschalk, Dean M. Wingerchuk. West Sussex, UK: Wiley Blackwell; 2015:13-20.

### Ethical issues in psychiatry (including gender and LGBT)

Alpert S: **The SPECTer of commercial neuroimaging**. *AJOB Neurosci* 2012, **3**(4):56-58. doi:10.1080/21507740.2012.721450.

Anderson JA, Illes J: **Neuroimaging and mental health: drowning in a sea of acrimony**. *AJOB Neurosci* 2012, **3**(4):42-43. doi:10.1080/21507740.2012.721454.

Appelbaum PS: **Ethical challenges in the primary prevention of schizophrenia.** *Schizophr Bull* 2015, **41**(4):773-775. doi:10.1093/schbul/sbv053.

Armon E, Kohls NB, Giordano J: **On the viability of neurotechnology and mind-body methods in pediatric mental health: perspectives on integrating new tools to complement old techniques**. *Eur J Integ Med* 2016, **8**(2):137-140. doi:10.1016/j.eujim.2015.12.011.

Barry LK: **Ethical issues in psychiatric research**. *Psychiatr Clin North Am* 2009, **32**(2):381-394. doi:10.1016/j.psc.2009.02.003.

Baylis F, Downie J: **Drilling down in neuroethics**. *Bioethics* 2009, **23**(6):iii-iv. doi:10.1111/j.1467-8519.2009.01730.x.

Belitz J, Bailey RA: **Clinical ethics for the treatment of children and adolescents: a guide for general psychiatrists**. *Psychiatr Clin North Am* 2009, **32**(2):243-257. doi:10.1016/j.psc.2009.02.001.

Bell JA: **Preventing post-traumatic stress disorder or pathologizing bad memories?** *Am J Bioeth* 2007, **7**(9): 29-30. doi:10.1080/15265160701518540.

Bikson M, Bhaskar P, Giordano J: **The off-label use, utility and potential value of tDCS in the clinical care of particular neuropsychiatric conditions**. *J Law Biosci* 2016, **3**(3):642-646. doi:10.1093/jlb/lsw044.

Bloch S, Green S: **An ethical framework for psychiatry**. *Br J Psychiatry* 2006, **188:**7-12. doi:10.1192/bjp.188.1.7.

Bockting WO: **Vulnerability and resilience among gender-nonconforming children and adolescents: mental health professionals have a key role to play**. *J Am Acad Child Adolesc Psychiatry* 2016, **55**(6):441-443. doi:10.1016/j.jaac.2016.04.008.

Bott NT, Radke AE, Kiely T: **Ethical issues surrounding psychologists’ use of neuroscience in the promotion and practice of psychotherapy**. *Prof Psychol Res Pr* 2016, **47**(5): 321-329. doi:10.1037/pro0000103.

Brown T: **Narrative practice apart from truth**. *AJOB Neurosci* 2012, **3**(4):91-92. doi:10.1080/21507740.2012.722809.

Buchman DZ, Skinner W, Illes J: **Negotiating the relationship between addiction, ethics, and brain science**. *AJOB Neurosci* 2010, **1**(1):36-45. doi:10.1080/21507740903508609.

Cabral M et al.: **Removal of gender incongruence of childhood diagnostic category: a human rights perspective**. *Lancet Psychiatry* 2016, **3**(5):405-406. doi:10.1016/S2215-0366(16)30043-8.

Carpenter W**: Diagnosing rather than labeling young people with mental disorders: a response to Mittal et al.** *Bioethics* 2015, **29**(9):683. doi:10.1111/bioe.12179.

Carter A, Hall W: **Beyond the right to injectable heroin**. *AJOB Neurosci* 2010, **1**(1):48-49. doi:10.1080/21507740903504467.

Charland LC: **A madness for identity: psychiatric labels, consumer autonomy, and the perils of the internet**. *Philos Psychiatr Psychol* 2004*,* **11**(4)**:**335-349.

Chen DT, Shepherd LL: **When, why, and how to conduct research in child and adolescent psychiatry: practical and ethical considerations**. *Psychiatr Clin North Am* 2009, **32**(2):361-380. doi:10.1016/j.psc.2009.03.003.

Cheung EH: **A new ethics of psychiatry: neuroethics, neuroscience, and technology**. *J Psychiatr Pract* 2009, **15**(5):391-401. doi:10.1097/01.pra.0000361279.11210.14.

Coverdale J, Roberts LW: **Global challenges and ethics in protecting and promoting the interests of psychiatrically ill patients.** *Int Rev Psychiatry* 2010, **22**(3):229-234. doi:10.3109/09540261.2010.486828.

Crane JK: **“Who you talkin’ about?”: parallel truthiness concerns between autobiography and biography in bioethics**. *AJOB Neurosci* 2012, **3**(4):82-83. doi:10.1080/21507740.2012.721458.

Daley TC, Singhal N, Krishnamurthy V**: Ethical considerations in conducting research on autism spectrum disorders in low and middle income countries.** *J Autism Dev Disord* 2013, **43**(9):2002-2014. doi:10.1007/s10803-012-1750-2.

Dell ML: **Child and adolescent depression: psychotherapeutic, ethical, and related nonpharmacologic considerations for general psychiatrists and others who prescribe.** *Psychiatr Clin North Am* 2012, **35**(1):181-201. doi:10.1016/j.psc.2011.12.002.

Diamond M: **Developmental, sexual and reproductive neuroendocrinology: historical, clinical and ethical considerations**. *Front Neuroendocrinol* 2011, **32**(2):255-263. doi:10.1016/j.yfrne.2011.02.003.

Drescher J, Cohen-Kettenis PT, Reed GM: **Gender incongruence of childhood in the ICD-11: controversies, proposal, and rationale**. *Lancet Psychiatry* 2016, **3**(3):297-304. doi:10.1016/S2215-0366(15)00586-6.

Drescher J. **Queer diagnoses: parallels and contrasts in the history of homosexuality, gender variance, and the Diagnostic and Statistical Manual**. *Arch Sex Behav* 2010, **39**(2):427-460. doi:10.1007/s10508-009-9531-5.

Drescher J: **Queer diagnoses revisited: the past and future of homosexuality and gender diagnoses in DSM and ICD**. *Int Rev Psychiatry* 2015, **27**(5):386-395. doi:10.3109/09540261.2015.1053847.

Farah MJ, Wolpe PR: **Monitoring and manipulating brain function: new neuroscience technologies and their ethical implications**. *Hastings Cent Rep* 2004, **34**(3):35-45.

Farah MJ, Gillihan SJ: **The puzzle of neuroimaging and psychiatric diagnosis: technology and nosology in an evolving discipline**. *AJOB Neurosci* 2012, **3**(4):31-41. doi:10.1080/21507740.2012.713072.

Fisher CE: **Neuroimaging and validity in psychiatric diagnosis**. *AJOB Neurosci* 2012, **3**(4):50-51. doi:10.1080/21507740.2012.721471.

Fitzpatrick SJ: **The telling moment: narrative as a discursive act**. *AJOB Neurosci* 2012, **3**(4):80-81. doi:10.1080/21507740.2012.721452.

Fuchs T: **Ethical issues in neuroscience**. *Curr Opin Psychiatry* 2006, **19**(6):600-607. doi:10.1097/01.yco.0000245752.75879.26.

Gibson S, Benson O, Brand SL: **Talking about suicide: confidentiality and anonymity in qualitative research**. *Nurs Ethics* 2013, **20**(1):18-29. doi:10.1177/0969733012452684.

Gillett GR: **The subjective brain, identity, and neuroethics**. *Am J Bioeth* 2009, **9**(9):5-13. doi:10.1080/15265160903090058.

Giordano J: **On the implications of changing constructs of pain and addiction disorders in the DSM-5: language games, ethics and action.** *IJLHE* 2011, **7**(1):1-8.

Giordano J: **Neuroimaging in psychiatry: approaching the puzzle as a piece of the bigger picture(s)**. *AJOB Neurosci* 2012, **3**(4):54-56. doi:10.1080/21507740.2012.721469.

Greely HT: **Direct brain interventions to “treat” disfavored human behaviors: ethical and social issues**. *Clin Pharmacol Ther* 2012, **91**(2):163-165. doi:10.1038/clpt.2011.292.

Greenberg D, Strous RD: **Ethics and the psychiatry journal editor: responsibilities and dilemmas.** *Isr J Psychiatry Relat Sci* 2014, **51**(3):204-210.

Gupta M: **Does evidence-based medicine apply to psychiatry?** *Theor Med Bioeth* 2007, **28**(2): 103-120. doi:10.1007/s11017-007-9029-x.

Hall W: **Feeling “better than well.”** *EMBO Rep* 2004, **5**(12):1105-1109. doi:10.1038/sj.embor.7400303.

Hens K, Peeters H, Dierickz K: **The ethics of complexity: genetics and autism, a literature review.** *Am J Med Genet B Neuropsychiatr Genet* 2016, **171B**(3):305-316. doi:10.1002/ajmg.b.32432.

Hoop JG, Smyth AC, Roberts LW: **Ethical issues in psychiatric research on children and adolescents.** *Child Adolesc Psychiatr Clin N Am* 2008, **17**(1):127-148. doi:10.1016/j.chc.2007.07.003.

Hoop JG, Spellecy R: **Philosophical and ethical issues at the forefront of neuroscience and genetics: an overview for psychiatrists**. *Psychiatr Clin North Am* 2009, **32**(2):437-449. doi:10.1016/j.psc.2009.03.004.

Horwitz AV, Wakefield JC: **Should screening for depression among children and adolescents be demedicalized?** *J Am Acad Child Adolesc Psychiatry* 2009, **48**(7):683-687. doi: 10.1097/CHI.0b013e3181a5e3ad.

Herrera-Ferrá K, Giordano J: **Re-classifying recurrent violent behavior? considerations, caveats and neuroethical concerns for psychiatry and social engagement**. *Acta Psychopathol* 2016, **2**:1. doi:10.4172/2469-6676.100032.

Jotterand F, Giordano J: **Transcranial magnetic stimulation, deep brain stimulation and personal identity: ethical questions, and neuroethical approaches for medical practice**. *Int Rev Psychiatry* 2011, **23**(5):476-485. doi:10.3109/09540261.2011.616189.2011.616189.

Kalis A: **Narrative truth and cases of delusion**. *AJOB Neurosci* 2012, **3**(4):87-89. doi:10.1080/21507740.2012.721457.

Katz CL, Lahey TP, Campbell HT: **An ethical framework for global psychiatry**. *Ann Glob Health* 2014, **80**(2):146-151. doi:10.1016/j.aogh.2014.04.002.

Koelch M, Fegert JM: **Ethics in child and adolescent psychiatric care: an international perspective.** *Int Rev Psychiatry* 2010, **22**(3):258-266. doi:10.3109/09540261.2010.485979.

Lavazza A: **Erasing traumatic memories: when context and social interests can outweigh personal autonomy**. *Philos Ethics Humanit Med* 2015,**10**:3. doi:10.1186/s13010-014-0021-6.

Lee G: **Ethical issues in behavioral neuroscience: ethics of human research in behavioral neuroscience: overview of Section II**. *Curr Top Behav Neurosci* 2015, **19**:135-136. doi:10.1007/7854_2014_343.

Lingiardi V, Nardelli N, Tripodi E: **Reparative attitudes of Italian psychologists toward lesbian and gay clients: theoretical, clinical, and social implications**. *Prof Psychol Res Pr* 2015, **46**(2):132-139. doi:10.1037/pro0000016.

Loi M: **Normal functioning and public reason**. *Camb Q Healthc Ethics* 2013, **22**(2):136-145. doi:10.1017/S0963180112000515.

Matthews S, Kennett J: **Lying, narrative, and truth shareability**. *AJOB Neurosci* 2012, **3**(4):86-87. doi:10.1080/21507740.2012.721468.

Mittal VA, Dean DJ, Mittal J, Saks ER: **Ethical, legal, and clinical considerations when disclosing a high-risk syndrome for psychosis**. *Bioethics* 2015, **29**(8):543-556. doi:10.1111/bioe.12155.

Mosley PE, Marsh R, Carter A: **Deep brain stimulation for depression: Unterrainer Mscientific issues and future directions.** *Aust N Z J Psychiatry* 2015, **4** (11):967-978. doi:10.1177/0004867415599845.

Northoff G: **Neuroscience of decision making and informed consent: an investigation in neuroethics**. *J Med Ethics* 2006, **32**(2):70-73. doi:10.1136/jme.2005.011858.

Northoff G: **What is neuroethics? empirical and theoretical neuroethics**. *Curr Opin Psychiatry* 2009, **22**(6):565-569. doi:10.1097/YCO.0b013e32832e088b.

Parmigiani G et al.: **Decisional capacity to consent to clinical research involving placebo in psychiatric patients.** *J Forensic Sci* 2016, **61**(2):388-393. doi:10.1111/1556-4029.13000.

Patil T, Giordano J: **On the ontological assumptions of the medical model of psychiatry: philosophical considerations and pragmatic tasks**. *Philos Ethics Humanit Med* 2010, **5**:3. doi:10.1186/1747-5341-5-3.

Radden J: **Notes towards a professional ethics for psychiatry**. *Aust N Z J Psychiatry* 2002, **36**(1)**:**52-59. doi:10.1046/j.1440-1614.2002.00989.x.

Ramos RT: **The conceptual limits of neuroimaging in psychiatric diagnosis**. *AJOB Neurosci* 2012, **3**(4):52-53. doi:10.1080/21507740.2012.721856.

Remschmidt H: **Psychopharmacological treatments in children and adolescents: adequate use or abuse?** *World Psychiatry* 2013, **12**(2):135-136. doi:10.1002/wps.20036.

Rich BA: **My story, my self: the pathologizing of personal identity**. *AJOB Neurosci* 2012, **3**(4):89-91. doi:10.1080/21507740.2012.721456.

Roskies AL: **Neuroethics beyond genetics: despite the overlap between the ethics of neuroscience and genetics, there are important areas where the two diverge**. *EMBO Rep* 2007, **8**:S52-S56. doi:10.1038/sj.embor.7401009.

Salles A: **Neuroethics in a “psy” world: the case of Argentina**. *Camb Q Healthc Ethics* 2014, **23**(3):297-307. doi:10.1017/S096318011300090X.

Sample M: **Evolutionary, not revolutionary: current prospects for diagnostic neuroimaging**. *AJOB Neurosci* 2012, **3**(4):46-48. doi:10.1080/21507740.2012.721461.

Satel S, Lilienfeld SO: **Singing the brain disease blues**. *AJOB Neurosci* 2010, **1**(1):46-47. doi:10.1080/21507740903504368.

Schechtman M: **Making the truth: self-understanding, self-constitution, neuroscience, and narrative**. *AJOB Neurosci* 2012, **3**(4):75-76. doi:10.1080/21507740.2012.721453.

Song SJ: **An ethical approach to life-long learning: implications for global psychiatry.** *Acad Psychiatry* 2011, **35**(6):391-396. doi:10.1176/appi.ap.35.6.391.

Stier M, Muders S, Rüther M, Schöne-Seifert B: **Biologismus-kontroversen: ethische implikationen für die psychiatrie [biologism controversy: ethical implications for psychiatry]**. *Nervenarzt* 2013, **84**(10):1165-1174. doi:10.1007/s00115-013-3736-5.

Strous RD: **Ethical considerations in clinical training, care and research in psychopharmacology**. *Int J Neuropsychopharmacol* 2011, **14**(3):413-424. doi:10.1017/S1461145710001112.

Suárez Richards M: **Psiquiatría y neuroética [psychiatry and neuroethics]**. *Vertex* 2013, **24**(109):233-240.

Synofzik M: **Intervenieren in der neuronalen basis der persönlichkeit: eine praxisorientierte ethische analyse der neuropharmakologie und der tiefen hirnstimulation [Intervening in the neural basis of one's personality: a practice-oriented ethical analysis of neuropharmacology and deep-brain stimulation]**. *Dtsch Med Wochenschr* 2007, **132**(50):2711-2713. doi:10.1055/s-2007-993124.

Takagi M: **精神医学的治療としての脳深部刺激の安全性と神経学的考察 [safety and neuroethical consideration of deep brain stimulation as a psychiatric treatment]**. *Brain Nerve* 2009, **61**(1):33-40.

Thachuk AK: **Stigma and the politics of biomedical models of mental illness.** *Int J Fem Approaches Bioeth* 2011, **4**(1):140-163. doi:10.2979/intjfemappbio.4.1.140.

Thong IS et al.: **Therapeutic misconception in psychiatry research: a systematic review.** *Clin Psychopharmacol Neurosci* 2016, **14**(1):17-25. doi:10.9758/cpn.2016.14.1.17.

Tsao CI, Layde JB, Roberts LW: **A review of ethics in psychiatric research**. *Curr Opin Psychiatry* 2008, **21**(6):572-577. doi:10.1097/YCO.0b013e32830aba23.

Umbach R, Berryessa CM, Raine A: **Brain imaging research on psychopathy: implications for punishment, prediction, and treatment in youth and adults.** *J Crim Jus* 2015, **43**(4):295-306. doi:10.1016/j.jcrimjus.2015.04.003.

Unterrainer M, Oduncu FS: **The ethics of deep brain stimulation (DBS).** *Med Health Care Philos* 2015, **18**(4):475-485. doi:10.1007/s11019-015-9622-0.

Van Niekerk AA: **Biomedical enhancement and the pursuit of mastery and perfection: a critique of the views of Michael Sandel**. *S Afr J Philos* 2014, **33**(2):155-165.

Vaz M, Srinivasan K: **Ethical challenges & dilemmas for medical health professionals doing psychiatric research.** *Indian J Med Res* 2014, **139**(2):191-193.

Vuilleumier P, Sander D, Baertschi B: **Changing the brain, changing the society: clinical and ethical implications of neuromodulation techniques in neurology and psychiatry**. *Brain Topogr* 2014, **27**(1):1-3. doi:10.1007/s10548-013-0325-7.

Walker MJ: **Neuroscience, self-understanding, and narrative truth**. *AJOB Neurosci* 2012, **3**(4):63-74. doi:10.1080/21507740.2012.712603.

Wolpe PR: **Ethics and social policy in research on the neuroscience of human sexuality**. *Nat Neurosci* 2004, **7**(10):1031-1033. doi:10.1038/nn1324.

Wright MT, Roberts LW: **A basic decision-making approach to common ethical issues in consultation-liaison psychiatry**. *Psychiatr Clin North Am* 2009, **32**(2):315-328. doi:10.1016/j.psc.2009.03.001.

Wurzman R, Giordano J: **Differential susceptibility to plasticity: a “missing link” between gene-culture coevolution and neuropsychiatric spectrum disorders?** *BMC Med* 2012, **10**:37. doi:10.1186/1741-7015-10-37.

Zohny H: **Enhancement, disability and the riddle of the relevant circumstances**. *J Med Ethics* 2016, **42**(9):605-610. doi:10.1136/medethics-2015-103229.

Books

Dogan H: *MIS-UN-TRUE Informed Consent: Focus on Informed Consent and Ethical Requirements for Clinical Trials of Neuroscience and Psychiatry*. Saarbrücken: Lambert Academic Publishing; 2015.

Elliot C: *Better Than Well: American Medicine Meets the American Dream.* New York: W.W.Norton; 2003.

Elliot C: Chambers T: *Prozac as a Way of Life*. Chapel Hill, NC: University of North Carolina Press; 2004.

Fulford KWM, Thornton T, Graham G: *Oxford Textbook of Philosophy and Psychiatry*. New York: Oxford University Press; 2006.

Gupta M: *Is Evidence-based Psychiatry Ethical?* Oxford: Oxford University Press; 2014.

Healy D: *Let Them Eat Prozac: The Unhealthy Relationship Between the Pharmaceutical Industry and Depression*. New York: New York University Press; 2004.

Lee G, Illes J, Ohl F: *Ethical Issues in Behavioral Neuroscience*. Heidelberg: Springer; 2015.

Phillips J: *Philosophical Perspectives on Technology and Psychiatry*. Oxford: Oxford University Press; 2009.

Sadler JZ, van Staden W, Fulford KWM: *The Oxford Handbook of Psychiatric Ethics*. Oxford: Oxford University Press; 2015.

Sahakian BJ, LaBuzetta JN: *Bad Moves: How Decision Making Goes Wrong, and the Ethics of Smart Drugs*. Oxford: Oxford University Press; 2013.

**Book Chapters**

Bluhm R, Raczek M, Broome, MR, Wall MB: **Ethical issues in brain imaging in psychiatry**. In *Oxford Handbook of Psychiatric Ethics*. Edited by John A. Sadler, Werdie van Staden, K.W.M. Fulford. Oxford: Oxford University Press; 2015:1109-1125.

Conrad P, Horwitz A: **Marketing of neuropsychiatric illness and enhancement.** In *Neuroethics in Practice.* Edited by Anjan Chatterjee, Martha J. Farah. New York: Oxford University Press; 2013:46-56.

Drescher J: **Ethical issues in treating LGBT patients**. In *Oxford Handbook of Psychiatric Ethics*. Edited by John A. Sadler, Werdie van Staden, K.W.M. Fulford. Oxford: Oxford University Press; 2015:180-192.

Glannon W: **Pharmacological and psychological interventions.** In his *Bioethics and the Brain.* New York: Oxford University Press; 2007:76-115.

Glannon W: **Psychiatric neuroethics I: deep brain stimulation and lesioning**. In *Oxford Handbook of Psychiatric Ethics*. Edited by John A. Sadler, Werdie van Staden, K.W.M. Fulford. Oxford: Oxford University Press; 2015:1191-1206.

Glannon W: **Psychiatric neuroethics II: less invasive and non-invasive techniques**. In *Oxford Handbook of Psychiatric Ethics*. Edited by John A. Sadler, Werdie van Staden, K.W.M. Fulford. Oxford: Oxford University Press; 2015:1207-1227.

Hansen JL: **A virtue-based approach to neuro-enhancement in the context of psychiatric practice**. In *Oxford Handbook of Psychiatric Ethics*. Edited by John A. Sadler, Werdie van Staden, K.W.M. Fulford. Oxford: Oxford University Press; 2015:1228-1249.

Kleinbub JR, Zidarich S: **Are near-death-experience memories real? ethical implications of a neuropsychological study**. In *Frontiers in Neuroethics: Conceptual and Empirical Advances*. Edited by Andrea Lavazza. Newcastle upon Tyne : Cambridge Scholars Publishing; 2016:135-156.

Radden J: **Virtue ethics as professional ethics; the case of psychiatry**. In *Working Virtue: Virtue Ethics and Contemporary Moral Problems.* New York: Oxford University Press; 2007:113-134.

### Ethical issues in anesthesiology/pain medicine (including addiction)

Brenner GJ, Kueppenbender K, Mao J, Spike J: **Ethical challenges and interventional pain medicine.** *Curr Pain Headache Rep* 2012, **16**(1):1-8. doi:10.1007/s11916-011-0242-y.

Carter D, Sendziuk P, Eliott JA, Braunack-Mayer A: **Why is pain still under-treated in the emergency department? two new hypotheses**. *Bioethics* 2016, **30**(3):195-202. doi:10.1111/bioe.12170.

Compagnone C, Tagliaferri F, Allegri M, Fanelli G: **Ethical issues in pain and omics research: some points to start the debate**. *Croat Med J* 2014, **55**(1):1-2. doi:10.3325/cmj.2014.55.1.

Compton P, Weaver MF: **Responsible opioid use**. *J Pain Palliat Care Pharmacother* 2015, **29**(2):166-168. doi:10.3109/15360288.2015.1037522.

da Cunha BF: **Ethics and undertreatment of pain in patients with a history of drug abuse**. *Medsurg Nurs* 2015, **24**(1):Suppl 4-7, 16.

Demertzi A et al.: **Pain perception in disorders of consciousness: neuroscience, clinical care, and ethics in dialogue**. *Neuroethics* 2013, **6**(1):37-50. doi:10.1007/s12152-011-9149-x.

Franklin GM: **Primum non nocere**. *Pain Med* 2013, **14**(5):617-618. doi:10.1111/pme.12120_2.

Garcia AM: **State laws regulating prescribing of controlled substances: balancing the public health problems of chronic pain and prescription painkiller abuse and overdose**. *J Law Med Ethics* 2012, **41**(Suppl 1):42-45. doi:10.1111/jlme.12037.

Giordano J: **Cassandra’s curse: interventional pain management, policy and preserving meaning against a market mentality**. *Pain Physician* 2006, **9**(3):167-169.

Giordano J: **Complementarity, brain-mind, and pain**. *Forsch Komplementmed* 2008, **15**(2):71-73. doi:10.1159/000121106.

Giordano J, Schatman ME: **A crisis in chronic pain care: an ethical analysis: part two: proposed structure and function of an ethics of pain medicine.** *Pain Physician* 2008, **11**(5): 589-595.

Giordano J, Schatman ME: **A crisis in chronic pain care: an ethical analysis: part three: toward an integrative, multi-disciplinary pain medicine built around the needs of the patient.** *Pain Physician* 2008, **11**(6): 775-784.

Giordano J, Engebretson JC, Benedikter R: **Culture, subjectivity, and the ethics of patient-centered pain care**. *Camb Q Healthc Ethics* 2009, **18**(1):47-56. doi:10.1017/S0963180108090087.

Giordano J, Schatman ME: **A ethical analysis of crisis in chronic pain care: facts, issues and problems in pain medicine: part 1.** *Pain Physician* 2008, **11**(4):483-490.

Giordano J, Schatman ME, Höver G: **Ethical insights to rapprochement in pain care: bringing stakeholders together in the best interest(s) of the patient.** *Pain Physician* 2009, **12**(4):E265-E275.

Giordano J: **Ethics of, and in, pain medicine: constructs, content, and contexts of application.** *Pain Physician* 2008, **11**(4):391-392.

Giordano J: **Maldynia: chronic pain as illness, and the need for complementarity in pain care**. *Forsch Komplementmed* 2008, **15**(5):277-281. doi:10.1159/000158572.

Giordano J: **Moral agency in pain medicine: philosophy, practice and virtue.** *Pain Physician* 2006, **9**(1):41-46.

Giordano J: **The neuroscience of pain, and a neuroethics of pain care.** *Neuroethics* 2010, **3**(1):89-94. doi:10.1007/s12152-009-9034-z.

Giordano J, Gomez CF, Harrison C: **On the potential role for interventional pain management in palliative care.** *Pain Physician* 2007, **10**(3):395-398.

Giordano J: **Pain and suffering: Körper and Leib, and the telos of pain care**. *Philos Psychiatr Psychol* 2013,**19**(4):279-283.

Giordano J, Abramson K, Boswell MV: **Pain assessment: subjectivity, objectivity, and the use of neurotechnology.** *Pain Physician* 2010, **13**(4):305-315.

Giordano J, Schatman ME, Benedikter R: **Pain care for a global community: part 2: ethics and economics in intersection**. *Pract Pain Manag* 2008, **8**(7):65-69.

Giordano J, Newman R, Boswell MV: **Pain medicine, biotechnology and market effects: tools, tekne and moral responsibility**. *Ethics Biol Eng Med* 2010, **1**(2):133-140. doi:10.1615/EthicsBiologyEngMed.v1.i2.50.

Giordano J, Boswell MV: **Pain, placebo, and nocebo: epistemic, ethical and practical issues**. *Pain Physician* 2005, **8**(4):331-333.

Giordano J: **Pain research: can paradigmatic expansion bridge the demands of medicine, scientific philosophy and ethics?** *Pain Physician* 2004, **7**(4):407-410.

Giordano J, Kohls NB: **Self, suffering and spirituality: the neuroscience of being, pain and spiritual experiences and practices**. *Mind Matter* 2008, **6**(2):179-191.

Giordano J: **Techniques, technology, and tekne: on the ethical use of guidelines in the practice of interventional pain medicine.** *Pain Physician* 2007, **10**(1**)**:1-5.

Giordano J, Benedikter R: **The shifting architectonics of pain medicine: toward ethical realignment of scientific, medical and market values for the emerging global community--groundwork for policy.** *Pain Med* 2011, **12**(3):406-414. doi:10.1111/j.1526-4637.2011.01055.x.

Goldberg DS, Rich BA: **Few benefits, likely harms: against universal random urine drug screening in pain management**. *Pain Med* 2014, **15**(12):2000-2001. doi:10.1111/pme.12604_3.

Gupta A, Giordano J: **On the nature, assessment, and treatment of fetal pain: neurobiological bases, pragmatic issues and ethical concerns**. *Pain Physician* 2007, **10**(4):525-532.

Heit HA: **Healthcare professionals and the DEA: trying to get back in balance**. *Pain Med* 2006, **7**(1):72-74. doi:10.1111/j.1526-4637.2006.00093.x

Knight C, Albertsen A: **Rawlsian justice and palliative care**. *Bioethics* 2015, **29**(8):536-542. doi:10.1111/bioe.12156.

Kotalik J: **Controlling pain and reducing misuse of opioids: ethical considerations**. *Can Fam Physician* 2012, **58**(4):381-385.

Loveless SE, Giordano J: **Neuroethics, painience, and neurocentric criteria for the moral treatment of animals**. *Camb Q Healthc Ethics* 2014, **23**(2):163-172. doi:10.1017/S0963180113000698.

Marcus BS, Venkat A: **Ethical pain management in the emergency department: the canary in the coal mine**. *Pain Manag* 2015, **5**(4):251-260. doi:10.2217/pmt.15.18.

McConnell D, Snoek A: **Narrating truths worth living: addiction narratives**. *AJOB Neurosci* 2012, **3**(4):77-78. doi:10.1080/21507740.2012.721459.

McGrew M, Giordano J: **Whence tendance? accepting the responsibility of care for the chronic pain patient**. *Pain Physician* 2009, **12**(3):483-485.

Niebrój LT, Jadamus-Niebrój D, Giordano J: **Toward a moral grounding of pain medicine: consideration of neuroscience, reverence, beneficence, and autonomy.** *Pain Physician* 2008, **11**(1):7-12.

Oliver J et al.: **American Society for Pain Management nursing position statement: pain management in patients with substance use disorders**. *J Addict Nurs* 2012, **23**(3):210-222. doi:10.1097/JAN.0b013e318271c123.

Olsen DP: **Ethical practice with patients in pain**. *Am J Nurs* 2016, **116**(1):57-60. doi:10.1097/01.NAJ.0000476172.74165.1c.

Payne R et al.: **A rose by any other name: pain contracts/agreements**. *Am J Bioeth* 2010, **10**(11):5-12. doi:10.1080/15265161.2010.519425.

Pentin PL: **Drug seeking or pain crisis? responsible prescribing of opioids in the emergency department**. *Virtual Mentor* 2013, **15**(5):410-415. doi:10.1001/virtualmentor.2013.15.5.ecas2-1305.

Peppin J: **Preserving beneficence**. *Pain Med* 2013, **14**(5):619. doi:10.1111/pme.12120_3.

Riganello F et al.: **Pain perception in unresponsive wakefulness syndrome may challenge the interruption of artificial nutrition and hydration: neuroethics in action**. *Front Neurol* 2016, **7**:202. doi:10.3389/fneur.2016.00202.

Robinson PJ, Rickard JA: **Ethical quandaries in caring for primary-care patients with chronic pain**. *Fam Syst Health* 2013, **31**(1):52-59. doi:10.1037/a0031952.

Robinson-Papp J, George MC: **Trust and the ethics of chronic pain management in HIV**. *J Assoc Nurses AIDS Care* 2015, **26**(5):509-513. doi:10.1016/j.jana.2015.05.007.

Venkat A et al.: **An ethical framework for the management of pain in the emergency department.** *Acad Emerg Med* 2013, **20**(7):716-723. doi:10.1111/acem.12158.

**Books:**

Benasayag M, Abtroun S: *L'éthique de la Souffrance [the ethics of suffering]*. Latresne: Bord de l'eau; 2002.

Bruder N, Bissonnette B, Ravussin P: *La Réanimation Neurochirurgicale [Neurosurgical Resuscitation]*. Paris: Springer; 2007.

Buonocore G, Bellieni CV: *Neonatal Pain: Suffering, Pain and Risk of Brain Damage in the Fetus and Newborn*. Milano: Springer-Verlag Italia; 2008.

Caenepeel D: *La Sédation Continue en Fin de Vie: Enjeux Ethiques [Sedation at the End of Life: Ethical Issues]*. Montréal: Médiaspaul; 2005.

Carter A, Hall W, Illes J: *Addiction Neuroethics: The Ethics of Addiction Neuroscience Research and Treatment*. London: Academic Press; 2012.

Cole BE: *Ethics in Pain Management and End-of-Life Care*. Glendale, CA: Audio-Digest Foundation; 2006.

Gallagher RM: *Insight and Responsibility: Ethics in Pain Medicine and Palliative Care*. London: Henry Stewart Talks; 2009.

George SK, Jung PG: *Cultural Ontology of the Self in Pain*. New Delhi: Springer; 2016.

Giordano J: *Maldynia: Multidisciplinary Perspectives on the Illness of Chronic Pain*. Boca Raton, FL: CRC Press; 2011.

Giordano JJ, Boswell MV: *Pain Medicine: Philosophy, Ethics and Policy*. Chicago: Linton Atlantic Books; 2009.

Giordano JJ: *Pain: Mind, Meaning, and Medicine: Collected Essays on the Philosophical and Ethical Dimensions of Practical Pain Management*. Glen Mills, PA: PPM Communications, Inc.; 2009.

Goldberg DS: *The Bioethics of Pain Management: Beyond Opioids*. New York: Routledge; 2014.

Green RM, Palpant NJ: *Suffering and Bioethics*. Oxford: Oxford University Press; 2014.

Grønstad A, Gustafsson H: *Ethics and Images of Pain*. New York: Routledge; 2012.

Husebø S, Klaschik E: *Palliativmedizin [Palliative Medicine]*. Berlin: Springer; 2009.

Loland S, Skirstad B, Waddington I: *Pain and Injury in Sport: Social and Ethical Analysis*. London: Routledge; 2006.

Meldrum M: *Opioids and Pain Relief: A Historical Perspective*. Seattle: IASP Press; 2003.

Morrissey MBQ: *Suffering Narratives of Older Adults: A Phenomenological Approach to Serious Illness, Chronic Pain, Recovery and Maternal Care*. New York: Routledge; 2015.

Schatman ME: *Ethical Issues in Chronic Pain Management*. New York: Informa Healthcare; 2007.

Schopenhauer A, Hollingdale RJ: *On the Suffering of the World*. London: Penguin Books; 2004.

van Norman GA, Jackson S, Rosenbaum SH, Palmer SK: *Clinical Ethics in Anesthesiology: A Case-Based Textbook*. New York: Cambridge University Press; 2011.

**Book Chapters:**

Giordano J: **From a neurophilosophy of pain to a neuroethics of pain care.** In *Scientific and Philosophical Perspectives in Neuroethics*. Edited by James J. Giordano, Bert Gordijn. New York: Cambridge University Press; 2010:172-189.

Hardcastle VG: **Minimally conscious states and pain: a different approach to patient ethics**. In *Finding Consciousness: The Neuroscience, Ethics, and Law of Severe Brain Damage*. Edited by Walter Sinnott-Armstrong. New York: Oxford University Press; 2016:207-225.

Hyman SE: **Addiction and responsibility**. In *Neuroethics in Practice*. Edited by Anjan Chatterjee, Martha J. Farah. New York: Oxford University Press; 2013:96-104.

Hyman SE: **The neurobiology of addiction: implications for the voluntary control of behavior.** In *Neuroethics: An Introduction with Readings*. Edited by Martha J. Farah. Cambridge, MA: MIT Press; 2010: 259-267.

## Neuroethics education/training

**Articles**

Aldrich R: **Nature, nurture and neuroscience: some future directions for historians of education**. *Paedagog Hist* 2014, **50**(6):852-860. doi:10.1080/00309230.2014.948011.

Bell E: **A room with a view of integrity and professionalism: personal reflections on teaching responsible conduct of research in the neurosciences**. *Sci Eng Ethics* 2015, **21**(2):461-469. doi:10.1007/s11948-014-9545-9.

Buchman DZ, Borgelt E, Illes J: **Core strategies for the development of a clinical neuroethics education program for medical residents in the clinical neurosciences**. *JEMH* 2009, **4**(2):1-6.

Busso D, Pollack C: **No brain left behind: consequences of neuroscience discourse for education**. *Learn Media Technol* 2015, **40**(2):168-186. doi:10.1080/17439884.2014.908908.

Fukushi T, Sakura O: **Neuroethics in Japan--current view and future visions**. *Brain Nerve* 2009, **61**(1):5-10.

Giordano J, Shook JR: **Minding brain science in medicine: on the need for neuroethical engagement for guidance of neuroscience in clinical contexts**. *Ethics Biol Eng Med* 2015, **6**(1-2):37-42. doi:10.1615/EthicsBiologyEngMed.2015015333.

Griffith JL: **Neuroscience and humanistic psychiatry: a residency curriculum**. *Acad Psychiatry* 2014, **38**(2):177-184. doi:10.1007/s40596-014-0063-5.

Han H: **How can neuroscience contribute to moral philosophy, psychology and education based on Aristotelian virtue ethics?** *Int J Ethics Edu* 2016, **1**(2):201-217. doi:10.1007/s40889-016-0016-9.

Hardiman M, Rinne L, Gregory E, Yarmolinskaya J: **Neuroethics, neuroeducation, and classroom teaching: where the brain sciences meet pedagogy**. *Neuroethics* 2012, **5**(2):135-143. doi:10.1007/s12152-011-9116-6.

Hook CJ, Farah MJ: **Neuroscience for educators: what are they seeking, and what are they finding?** *Neuroethics* 2013, **6**(2):331-341. doi:10.1007/s12152-012-9159-3.

Horvath JC, Donoghue GM: **A bridge too far -- revisited: reframing Bruer’s neuroeducation argument for modern science of learning practitioners**. *Front Psychol* 2016, **7**:377. doi:10.3389/fpsyg.2016.00377.

Hruby GG: **Three requirements for justifying an educational neuroscience**. *Br J Educ Psychol* 2012, **82**(Pt 1):1-23. doi:10.1111/j.2044-8279.2012.02068.x.

Illes J. **Interview with Judy Illes**. *Trends Neurosci* 2012, **35**(9):521-523. doi:10.1016/j.tins.2012.06.006.

Kalichman M, Sweet M, Plemmons D: **Standards of scientific conduct: disciplinary differences**. *Sci Eng Ethics* 2015, **21**(5):1085-1093. doi:10.1007/s11948-014-9594-0.

Kehagia AA et al.: **More education, less administration: reflections of neuroimagers' attitudes to ethics through the qualitative looking glass**. *Sci Eng Ethics* 2012, **18**(4):775-788. doi:10.1007/s11948-011-9282-2.

Knox R: **Mind, brain, and education: a transdisciplinary field**. *Mind Brain Educ* 2016, **10**(1):4-9. doi:10.1111/mbe.12102.

Lampe M: **Science, human nature, and a new paradigm for ethics education**. *Sci Eng Ethics* 2012, **18**(3):543-549. doi:10.1007/s11948-012-9373-8.

Langlais PJ: **Ethical decision making in the conduct of research: role of individual, contextual, and organizational factors: commentary on “science, human nature, and a new paradigm for ethics education”**. *Sci Eng Ethics* 2012, **18**(3):551-555. doi:10.1007/s11948-012-9371-x.

Lombera S, Fine A, Grunau RE, Illes J: **Ethics in neuroscience graduate training programs: views and models from Canada**. *Mind Brain Educ* 2010, **4**(1):20-27. doi:10.1111/j.1751-228X.2009.01079.x

Maxwell B, Racine E: **The ethics of neuroeducation: research, practice and policy.** *Neuroethics* 2012, **5**(2):101-103. doi:10.1007/s12152-012-9156-6.

Morein-Zamir S, Sahakian BJ: **Neuroethics and public engagement training needed for neuroscientists**. *Trends Cogn Sci* 2010, **14**(2):49-51. doi:10.1016/j.tics.2009.10.007.

Parra NH: **Neuroética y neuropolítica: sugerencias para la educación moral [neuroethics and neuropolitics: suggestions for moral education]**. *Anámnesis* 2011, **6**:62-66.

Rommelfanger KS, Johnson LSM: **What lies ahead for neuroethics scholarship and education in light of the Human Brain Project?** *AJOB Neurosci* 2015, **6**(1):1-3. doi:10.1080/21507740.2015.1000158.

Sahakian BJ, Morein-Zamir S: **Neuroscientists need neuroethics teaching**. *Science* 2009, **325**(5937):147. doi:10.1126/science.325_147a.

Sayre MM: **Teaching ethics informed by neuroscience**. *J Teach Soc Work* 2016, **36**(3):302-311. doi:10.1080/08841233.2016.1173616.

Secko DM, Burgess M, O’Doherty K: **Perspectives on engaging the public in the ethics of emerging biotechnologies: from salmon to biobanks to neuroethics**. *Account Res* 2008, **15**(4):283-302. doi:10.1080/08989620802388762.

Smeyers P: **Neurophilia: guiding educational research and the educational field?** *J Philos Educ* 2016, **50**(1):62-75. doi:10.1111/1467-9752.12173.

Sperduti A, Crivellaro F, Rossi PF, Bondioli L: **"Do octopuses have a brain?" knowledge, perceptions and attitudes towards neuroscience at school**. *PLoS One* 2012, **7**(10):e47943. doi:10.1371/journal.pone.0047943.

Walther G: **Ethics in neuroscience curricula: a survey of Australia, Canada, Germany, the UK, and the US.** *Neuroethics* 2013, **6**(2):343-351. doi:10.1007/s12152-012-9168-2.

Zocchi M, Pollack C: **Educational neuroethics: a contribution from empirical research.** *Mind Brain Educ* 2013, 7(1):56-62. doi:10.1111/mbe.12008.

**Books**

Chatterjee A, Farah MJ: *Neuroethics in Practice: Medicine, Mind, and Society.* Oxford: Oxford University Press; 2013.

Fischman W, Solomon B, Greenspan D, Gardner H: *Making Good: How Young People Cope with Moral Dilemmas at Work.* Cambridge, MA: Harvard University Press; 2004.

Illes, J: *Neuroethics: Defining the Issues in Theory, Practice and Policy*. Oxford: Oxford University Press; 2006.

Joldersma CW: *Neuroscience and Education: A Philosophical Appraisal*. New York: Routledge; 2016.

Organisation for Economic Co-operation and Development (OECD). *Understanding the Brain: The Birth of a Learning Science*. Paris: OECD; 2007.

Presidential Commission for the Study of Bioethical Issues (PCSBI). *Gray Matters: Topics at the Intersection of Neuroscience, Ethics, and Society (Vol 1).* Washington, DC: PCSBI; 2014.

**Book chapters**:

Ansari D: **Mind, brain, and education: a discussion of practical, conceptual, and ethical issues**. In *Handbook of Neuroethics*. Edited by Jens Clausen, Neil Levy. Dordrecht: Springer; 2015:1703-1719.

Brown TR, McCormick JB: **New directions in neuroscience policy**. In T*he Oxford Handbook of Neuroethics*. Edited by Judy Illes, Barbara J. Sahakian. Oxford: Oxford University; 2011:675-700.

Cooper P: **Education in the age of Ritalin**. In *The New Brain Sciences: Perils and Prospects*. Edited by Dai Reese, Steven Rose. Cambridge: Cambridge University Press; 2005:249-262.

Kalbfleisch ML: **Is the use of neurotechnology in education an enablement, treatment, or enhancement?** In *Neurotechnology: Premises, Potential, and Problems*. Edited by James Giordano. Boca Raton: CRC Press; 2012:37-46.

Minehata M, Walther G: **Biosecurity education and awareness in neuroscience**. In *Handbook of Neuroethics*. Edited by Jens Clausen, Neil Levy. Dordrecht: Springer; 2015:1773-1783.

Morselli PL: **Views on developments in brain science from a societal perspective: statement**. In *Connecting Brains and Society (International Workshop)*. Edited by Peter Raeymaekers, Karin Rondia, Marjan Slob. Brussels: King Baudouin Foundation; 2004:87-89.

Sheridan K, Zinchenko E, Gardner H: **Neuroethics in education**. In *Neuroethics: Defining the Issues in Theory, Practice and Policy*. Edited by Judy Illes. Oxford: Oxford University Press; 2006:265-275.

Stein Z, Bruno DC, Hinton C: **Ethical issues in educational neuroscience: raising children in a brave new world**. In T*he Oxford Handbook of Neuroethics*. Edited by Judy Illes, Barbara J. Sahakian. Oxford: Oxford University; 2011:803-822.

Tairyan K, Frank E: **Learning about neuroethics through health sciences online: a model for global dissemination**. In T*he Oxford Handbook of Neuroethics*. Edited by Judy Illes, Barbara J. Sahakian. Oxford: Oxford University; 2011:879-892.

## Neuroethics and law

**Articles**

Aggarwal NK, Ford E: **The neuroethics and neurolaw of brain injury**. *Behav Sci Law* 2013, **31**(6):789-802. doi:10.1002/bsl.2086.

Aggarwal NK: **Neuroimaging, culture, and forensic psychiatry.** *J Am Acad Psychiatry Law* 2009, **37**(2):239-244.

Aharoni E, Funk C, Sinnott-Armstrong W, Gazzaniga M: **Can neurological evidence help courts assess criminal responsibility? lessons from law and neuroscience**. *Ann N Y Acad Sci* 2008, 1124:145-160. doi:10.1196/annals.1440.007.

Aharoni E et al.: **Neuroprediction of future rearrest**. *Proc Natl Acad Sci U S A* 2013, **110**(15):6223-6228. doi:10.1073/pnas.1219302110.

Aronson JD: **The law’s use of brain evidence**. *Annu Rev Law Soc Sci* 2010, **6**:93-108. doi:10.1146/annurev-lawsocsci-102209-152948.

Balmakund Z: **The realities of neurolaw: a composition of data & research**. *U St Thomas JL & Pub Pol* 2015, **9**(2):189-207.

Beecher-Monas E, Garcia-Rill E: **Overselling images: fMRI and the search for truth**. *J Marshall Law Rev* 2015, **48**(3):651-692.

Bourgeois ML: **Neurosciences et neurodroit [Neurosciences and law]**. *Encephale* 2015, **41**(5):383-384. doi:10.1016/j.encep.2015.09.004.

Berlin L: **Neuroimaging, expert witnesses, and ethics: convergence and conflict in the courtroom**. *AJOB Neurosci* 2014, **5**(2):3-8. doi:10.1080/21507740.2014.880089.

Brindley T, Giordano J: **Neuroimaging - correlation, validity, value and admissibility: Daubert – and reliability – revisited**. *AJOB Neurosci* 2014, **5**(2): 48-50. doi:10.1080/21507740.2014.884186.

Brooks JD: “**What any parent knows” but the Supreme Court misunderstands: reassessing neuroscience’s role in diminished capacity jurisprudence**. *New Crim L Rev* 2014, **17**(3):442-501. doi:10.1525/nclr.2014.17.3.442.

Bublitz JC, Merkel R: **Crimes against minds: on mental manipulations, harms and a human right to mental self-determination**. *Crim L Philos* 2014, **8**(1):51-77. doi:10.1007/s11572-012-9172-y.

Buchanan A: **Mental capacity, legal competence and consent to treatment.** *J R Soc Med* 2004, **97**(9):415-420. doi:10.1258/jrsm.97.9.415.

Buckholtz JW et al.: **From blame to punishment: disrupting prefrontal cortex activity reveals norm enforcement mechanisms**. *Neuron* 2015, **87**(6):1369-1380. doi:10.1016/j.neuron.2015.08.023.

Catley P, Claydon L: **The use of neuroscientific evidence in the courtroom by those accused of criminal offenses in England and Wales**. *J Law Biosci* 2016, **2**(3):510-549. doi:10.1093/jlb/lsv025.

Chaffee EC: **The death and rebirth of codes of legal ethics: how neuroscientific evidence of intuition and emotion in moral decision making should impact the regulation of the practice of law**. *Geo J Legal Ethics* 2015, **28**(2):323-369.

Chandler JA: **The use of neuroscientific evidence in Canadian criminal proceedings**. *J Law Biosci* 2016, **2**(3):550-579. doi:10.1093/jlb/lsv026.

Choi Y et al.: **Detecting deception using neuroscience: a review of lie detection using functional magnetic resonance imaging**. *Korean J Biol Psychiatry* 2015, 22(3):109-112.

Church EJ: **Imaging’s insights into human violence**. *Radiol Technol* 2014, **85**(4):417-444.

Church DJ: **Neuroscience in the courtroom: an international concern.** *Wm Mary Law Rev* 2012, **53**(5):1826-1854.

Cohen AO, Casey BJ: **Rewiring juvenile justice: the intersection of developmental neuroscience and legal policy**. *Trends Cogn Sci* 2014, **18**(2):63-65. doi:10.1016/j.tics.2013.11.002.

De Amorim AR: **Equality and right to development as neuroethical concerns: assuring defendants’ rights**. *Am J Bioeth* 2008, **8**(1):28-30. doi:10.1080/15265160701828428.

de Kogel CH, Schrama WM, Smit M: **Civil law and neuroscience.** *Psychiatr Psychol Law* 2014, **21**(2):272-285. doi:10.1080/13218719.2013.808978.

Cohen AO et al.: **When is an adolescent an adult? assessing cognitive control in emotional and nonemotional contexts**. *Psychol Sci* 2016, **27**(4):549-562. doi:10.1177/0956797615627625.

Condlin R: **The “nature” of legal dispute bargaining**. *Cardozo J Conflict Resol* 2016, **17**(2):393-426.

Farah MJ: **Brain images, babies, and bathwater: critiquing critiques of functional neuroimaging**. *Hastings Cent Rep* 2014, **Spec No**:S19-S30. doi:10.1002/hast.295.

Farah MJ: **Neuroethics: the ethical, legal, and societal impact of neuroscience**. *Annu Rev Psychol* 2012, **63**:571-591. doi:10.1146/annurev.psych.093008.100438.

Farahany NA: **Neuroscience and behavioral genetics in US criminal law: an empirical analysis**. *J Law Biosci* 2016, **2**(3):485-509. doi:10.1093/jlb/lsv059.

Farisco M, Petrini C: **On the stand: another episode of neuroscience and law discussion from Italy**. *Neuroethics* 2014, **7**(2):243-245. doi: 10.1007/s12152-013-9187-7.

Fine C, Kennett J: **Mental impairment, moral understanding and criminal responsibility: psychopathy and the purposes of punishment**. *Int J Law Psychiatry* 2004, **27**(5):425-443. doi:10.1016/j.ijlp.2004.

Garland B, Glimcher PW: **Cognitive neuroscience and the law.** *Curr Opin Neurobiol* 2006, **16**(2):130-134. doi:10.1016/j.conb.2006.03.011.

Gaudet LM, Lushing JR, Kiehl KA: **Functional magnetic resonance imaging in court**. *AJOB Neurosci* 2014, **5**(2):43-45. doi:10.1080/21507740.2014.884192.

Giordano J, Kulkarni A, Farwell J: **Deliver us from evil? the temptation, realities and neuroethico-legal issues of employing assessment neurotechnologies in public safety initiatives.** *Theor Med Bioeth* 2014, **35**(1):73-89. doi:10.1007/s11017-014-9278-4.

Giordano J, Olds J: **On the interfluence of neuroscience, neuroethics and legal and social issues: the need for (N)ELSI.** *AJOB Neurosci* 2010, **1**(4):12-14. doi:10.1080/21507740.2010.515964.

Giordano J: **Physicians, payments, and practices: moral issues, public perceptions, and the Stark laws**. *Pain Physician* 2007, **10**(6):719-723.

Gkotsi GM, Gasser J: **Neuroscience in forensic psychiatry: from responsibility to dangerousness: ethical and legal implications of using neuroscience for dangerousness assessments.** *Int J Law Psychiatry* 2016, **46**:58-67. doi:10.1016/j.ijlp.2016.02.030.

Gkotsi GM, Moulin V, Gasser J: **Les neurosciences au Tribunal: de la responsibilité à la dangerosité, enjeux éthiques soulevés par la nouvelle loi Française**. [**Neuroscience in the courtroom: from responsibility to dangerousness, ethical issues raised by the new French law.]** *Encephale* 2015, **41**(5):385-393. doi:10.1016/j.encep.2014.08.014.

Glannon W: **The limitations and potential of neuroimaging in the criminal law**. *J Ethics* 2014, **18**(2):153-170. doi:10.1007/s10892-014-9169-y.

Glenn AL, Raine A: **Neurocriminology: implications for the punishment, prediction and prevention of criminal behaviour.** *Nat Rev Neurosci* 2014, **15**(1):54-63. doi:10.1038/nrn3640.

Glenn AL, Raine A: **Pyschopathy and instrumental aggression: evolutionary, neurobiological, and legal perspectives.** *Int J Law Psychiatry* 2009, **32**(4):253-258. doi:10.1016/j.ijlp.2009.04.002.

Granacher RP Jr, Sweet JJ, Felthous AR: **Introduction to this issue: traumatic brain injury**. *Behav Sci Law* 2013, **31**(6):683-685. doi:10.1002/bsl.2093.

Greely HT: **What if? the farther shores of neuroethics: commentary on "neuroscience may supersede ethics and law".** *Sci Eng Ethics* 2012, **18**(3):439-446. doi:10.1007/s11948-012-9391-6.

Greene J, Cohen J: **For the law, neuroscience changes nothing and everything.** *Philos Trans R Soc Lond B Biol Sci* 2004, **359**(1451):1775-1785. doi:10.1098/rstb.2004.1546.

Hauser LL: **Forensic implications of neuroscientific advancements.** *J Am Acad Psychiatry Law* 2016, **44**(2):193-197.

Hauser SL: **Advancing ethical neuroscience**. *Ann Neurol* 2015, **77**(5):735-737. doi:10.1002/ana.24411.

Henry S, Plemmons D: **Neuroscience, neuropolitics and neuroethics: the complex case of crime, deception and FMRI**. *Sci Eng Ethics* 2012, **18**(3):573-591. doi:10.1007/s11948-012-9393-4.

Horstkötter D et al.: **Neuroimaging in the courtroom: normative frameworks and consensual practices**. *AJOB Neurosci* 2014, **5**(2):37-39. doi:10.1080/21507740.2014.884190.

Hyman SE: **Emerging neurotechnologies for lie-detection: where are we now? an appraisal of Wolpe, Foster and Langleben’s “emerging neurotechnologies for lie-detection: promises and perils” five years later**. *Am J Bioeth* 2010, **10**(10):49-50. doi:10.1080/15265161.2010.527263.

Illes J: **A fish story? brain maps, lie detection, and personhood**. *Cerebrum* 2004, **6**(4):73-80.

Jones OD, Marois R, Farah MJ, Greely HT: **Law and neuroscience.** *J Neurosci* 2013, **33**(45):17624-17630. doi:10.1523/JNEUROSCI.3254-13.2013.

Jones OD, Wagner AD, Faigman DL, Raichle ME: **Neuroscientists in court.** *Nat Rev Neurosci* 2013, **14**(10):730-736. doi:10.1038/nrn3585.

Jungbauer WG, Bowman CW: **Daubert, Frye and DTI: hijacking the right to trial by jury**. *AJOB Neurosci* 2014, **5**(2):16-23. doi:10.1080/21507740.2014.885096.

Kalis A, Meynen G: **Mental disorder and legal responsibility: the relevance of stages of decision making.** *Int J Law Psychiatry* 2014, **37**(6):601-608. doi:10.1016/j.ijlp.2014.02.034.

Kolber AJ: **Two views of First Amendment thought privacy**. *U Pa J Const L* 2016, **18**:1381-1423.

Knabb JJ, Welsh RK, Ziebell JG, Reimer KS: **Neuroscience, moral reasoning, and the law**. *Behav Sci Law* 2009, **27**(2):219-236. doi:10.1002/bsl.854.

Kulynych J: **Legal and ethical issues in neuroimaging research: human subjects protection, medical privacy, and the public communication of research results.** *Brain Cogn* 2002, **50**(3):345-357. doi:10.1016/S0278-2626(02)00518-3.

Levy N: **Is neurolaw conceptually confused?** *J Ethics* 2014, **18**(2):171-185. doi:10.1007/s10892-014-9168-z.

Lippert-Rasmussen K: **Neuroprediction, truth-sensitivity, and the law**. *J Ethics* 2014, **18**(2):123-136. doi:10.1007/s10892-014-9162-5.

Macdonald M: **Some ethical issues in brain imaging**. *Cortex* 2011, **47**(10):1272-1274. doi:10.1016/j.cortex.2011.05.001.

Meegan DV: **Neuroimaging techniques for memory detection: scientific, ethical, and legal issues**. *Am J Bioeth* 2008, **8**(1):9-20. doi:10.1080/15265160701842007.

Meijer EH et al.: **Deception detection with behavioral, autonomic, and neural measures: conceptual and methodological considerations that warrant modesty**. *Psychophysiology* 2016, **53**(5):593-604. doi:10.1111/psyp.12609.

Meltzer CC et al.: **Guidelines for the ethical use of neuroimages in medical testimony: report of a multidisciplinary consensus conference**. *AJNR Am J Neuroradiol* 2014, **35**(4):632-637. doi:10.3174/ajnr.A3711.

Meynen G: **Neurolaw: neuroscience, ethics, and law: review essay.** *Ethical Theory Moral Pract* 2014, **17**(4):819-829. doi:10.1007/s10677-014-9501-4.

Meynen G: **A neurolaw perspective on psychiatric assessments of criminal responsibility: decision-making, mental disorder, and the brain.** *Int J Law Psychiatry* 2013, **36**(2):93-99. doi:10.1016/j.ijlp.2013.01.001.

Meynen G: **Neurolaw: recognizing opportunities and challenges for psychiatry.** *J Psychiatry Neurosci* 2016, **41**(1):3-5. doi:10.1503/jpn.150317.

Moore MS: **Stephen Morse on the fundamental psycho-legal error**. *Crim Law Philos* 2016, **10**(1):45-89. doi:10.1007/s11572-014-9299-0.

Morse SJ: **Brain imaging in the courtroom: the quest for legal relevance**. *AJOB Neurosci* 2014, **5**(2):24-27. doi:10.1080/21507740.2014.880090.

Morse SJ: **Brain overclaim syndrome and criminal responsibility: a diagnostic note.** *Ohio St J Crim L 2*006, **3**:397-412.

Morse SJ: **New neuroscience, old problems: legal implications of brain science.** *Cerebrum* 2004, **6**(4):81-90.

Morse SJ: **Psychopathy and criminal responsibility.** *Neuroethics* 2008, **1**(3):205-212. doi:10.1007/s12152-008-9021-9.

Murrow GB, Murrow R: **A hypothetical neurological association between dehumanization and human rights abuses**. *J Law Biosci* 2015, **2**(2):336-364. doi:10.1093/jlb/lsv015.

O’Connell K: **Unequal brains: disability discrimination laws and children with challenging behaviour.** *Med Law Rev* 2016, **24**(1):76-98. doi:10.1093/medlaw/fwv043.

Pallarés-Domínguez D, González-Esteban E: T**he ethical implications of considering neurolaw as a new power**. *Ethics Behav* 2016, **26**(3):252-266. doi:10.1080/10508422.2015.1012763.

Pickersgill M: **Connecting neuroscience and law: anticipatory discourse and the role of sociotechnical imaginaries.** *New Genet Soc* 2011, **30**(1):27-40. doi:10.1080/14636778.2011.552298.

Roginsky AB, Tsesis A: **Hate speech, volition, and neurology**. *J Law Biosci* 2016, **3**(1):174-177. doi:10.1093/jlb/lsv058.

Rose N: **“Screen and intervene”: governing risky brains**. *Hist Human Sci* 2010, **23**(1):79-105. doi:10.1177/0952695109352415.

Sade RM: **Introduction: brain science in the 21st century: clinical controversies and ethical and legal implications**. *J Law Med Ethics* 2014, **42**(2):124-127. doi:10.1111/jlme.12125.

Salmanowitz N: **The case for pain neuroimaging in the courtroom: lessons from deception detection**. *J Law Biosci* 2015, **2**(1):139-148. doi:10.1093/jlb/lsv003.

Schleim S: **Brains in context in the neurolaw debate: the examples of free will and “dangerous” brains.** *Int J Law Psychiatry* 2012, **35**(2):104-111. doi:10.1016/j.ijlp.2012.01.001.

Scott TR: **Neuroscience may supersede ethics and law**. *Sci Eng Ethics* 2012, **18**(3):433-437. doi:10.1007/s11948-012-9351-1.

Scurich N, Shniderman A: **The selective allure of neuroscientific explanations**. *PLoS One* 2014, **9**(9):e107529. doi:10.1371/journal.pone.0107529.

Shats K, Brindley T, Giordano J: **Don’t ask a neuroscientist about phases of the moon: applying appropriate evidence law to the use of neuroscience in the courtroom**. *Camb Q Healthc Ethics* 2016, **25**(4):712-725. doi:10.1017/S0963180116000438.

Sgarbi A: **The mystery of freedom and neurolaw**. *Beijing Law Rev* 2015, **6**:133-146. doi:10.4236/blr.2015.62014.

Shen FX: **Neuroscience, mental privacy, and the law.** *Harv J Law Public Policy* 2013, **36(2)**:654-713.

Sinnott-Armstrong WP: **Neurolaw and consciousness detection**. *Cortex* 2011, **4**7(10):1246-1247. doi:10.1016/j.cortex.2011.04.021.

Sirgiovanni E, Corbellini G, Caporale C: **A recap on Italian neurolaw: epistemological and ethical issues**. *Mind Soc* 2016, online only. doi:10.1007/s11299-016-0188-1.

Smith SR: **Neuroscience, ethics and legal responsibility: the problem of the insanity defense: commentary on "the ethics of neuroscience and the neuroscience of ethics: a phenomenological-existential approach".** *Sci Eng Ethics* 2012, **18**(3):475-481. doi:10.1007/s11948-012-9390-7.

Stoller SE, Wolpe PR: **Emerging neurotechnologies for lie detection and the Fifth Amendment**. *Am J Law Med* 2007, **33**(2-3):359-375. doi:10.1177/009885880703300210.

Tovino S: **The impact of neuroscience on health law.** *Neuroethics* 2008, **1**(2): 101-117. doi:10.1007/s12152-008-9010-z.

Wachbroit R: **The prospects for neuro-exceptionalism: transparent lies, naked minds**. *Am J Bioeth* 2008, **8**(1):3-8. doi:10.1080/15265160701828576.

Wolf SM: **Neurolaw: the big question**. *Am J Bioeth* 2008, **8**(1):21-22. doi:10.1080/15265160701828485.

Wolpe PR, Foster KR, Langleben DD: **Emerging neurotechnologies for lie-detection: promises and perils**. *Am J Bioeth* 2010, **10**(10):40-48. doi:10.1080/15265161.2010.519238.

Young G: **Causality in criminal forensic and in civil disability cases: legal and psychological comparison**. *Int J Law Psychiatry* 2015, **42-43**:113-120. doi:10.1016/j.ijlp.2015.08.015.

**Books**

Benforado A: *Unfair: The New Science of Criminal Injustice*. New York: Broadway Books; 2015.

Bernat JL, Beresford HR: *Ethical and Legal Issues in Neurology.* Edinburgh: Elsevier; 2013.

Dennett DC, Max Weber Programme: *“My Brain Made Me Do It”: (When Neuroscientists Think They Can Do Philosophy*. Florence: European University Institute; 2011.

Di Giovine O: *Diritto penale e neuroetica: atti del convegno, 21-22 maggio 2012, Università degli studi di Foggia [criminal law and neuroethics: the conference proceedings, 21 to 22 May 2012, University of Foggia studies]*. Padova: Casa Editrice Dott Antonio Milani; 2013.

Farahany NA: *The Impact of Behavioral Sciences on Criminal Law*. New York: Oxford University Press; 2009.

Garland B: *Neuroscience and the Law: Brain, Mind and the Scales of Justice*. New York: Dana Press, and Washington, DC: AAAS; 2004.

Koivula N et al.: *Neurolaw*. Maastricht, Netherlands: Maastricht University; 2014.

Morse SJ, Roskies AL: *A Primer on Criminal Law and Neuroscience*. New York: Oxford University Press; 2013.

Pardo MS, Patterson DM: *Minds, Brains, and Law: The Conceptual Foundations of Law and Neuroscience*. New York: Oxford University Press; 2013.

Patterson D, Pardo MS: *Philosophical Foundations of Law and Neuroscience*. Oxford: Oxford University Press; 2016.

President’s Council on Bioethics. *An Overview of the Impact of Neuroscience Evidence in Criminal Law*. Washington, DC: President’s Council on Bioethics; 2004.

Singh I, Sinnott-Armstrong WP, Savulescu J: *Bioprediction, Biomarkers, and Bad Behavior: Scientific, Legal, and Ethical Challenges*. New York: Oxford University Press; 2013.

Spranger TM: *International Neurolaw: A Comparative Analysis*. New York: Springer; 2012.

Vallabhajosula B: *Murder in the Courtroom: The Cognitive Neuroscience of Violence*. New York: Oxford University Press; 2015.

**Book Chapters**

Baum ML: **Reduced responsibility: distinguishing conditions in which biomarkers properly reduce legal responsibility.** In his *The Neuroethics of Biomarkers: What the Development of Bioprediction Means for Moral Responsibility, Justice, and the Nature of Mental Disorder*. New York: Oxford University Press; 2016:117-142.

Bernat J: **Clinical ethics and law.** In his *Ethical Issues in Neurology.* 3rd edition. Philadelphia: Lippincott Williams & Wilkins; 2008: 81-108.

Bublitz JC: **Drugs, enhancements, and rights: ten points for lawmakers to consider.** In *Cognitive Enhancement: Ethical and Policy Implications in International Perspectives.* Edited by Fabrice Jotterand and Veljko Dubljević. New York: Oxford University Press; 2016:309-328.

Chandler JA, Dodek AM: **Cognitive enhancement in the courtroom: the ethics of pharmacological enhancement of judicial cognition.** In *Cognitive Enhancement: Ethical and Policy Implications in International Perspectives.* Edited by Fabrice Jotterand and Veljko Dubljević. New York: Oxford University Press; 2016: 329-345.

Chandler J: **Mind, brain, and law: issues at the intersection of neuroscience, personal identity, and the legal system**. In *Handbook of Neuroethics*. Edited by Jens Clausen, Neil Levy. Dordrecht: Springer Netherlands; 2015:441-458.

Coppola F: **Innovating witness testimony with neuroscience-based lie detection: a hypothetical normative framework**. In *The Challenge of Innovation in Law: The Impact of Technology and Science on Legal Studies and Practice*. Edited by Amedeo Santosuosso, Oliver R. Goodenough, Marta Tomasi. Pavia, Italy: European Centre for Law, Science and New Technologies; 2015:145-166.

Farwell JP: **Issues of law raised by developments and use of neuroscience and neurotechnology in national security and defense**. In *Neurotechnology in National Security and Defense: Practical Considerations, Neuroethical Concerns*. Edited by James Giordano. Boca Raton: CRC Press; 2015:133-165.

Fins JJ, Pohl B: **Guardianship and the injured brain: representation and the rights of patients and families**. In *Finding Consciousness: The Neuroscience, Ethics, and Law of Severe Brain Damage*. Edited by Walter Sinnott-Armstrong. New York: Oxford University Press; 2016:246-258.

Glannon W: **What neuroscience can (and cannot) tell us about criminal responsibility**. In his *Brain, Body, and Mind: Neuroethics with a Human Face*. New York: Oxford University Press; 2011:72-92.

Greely HT: **The social effects of advances in neuroscience: legal problems, legal perspectives**. In *Neuroethics: Defining the Issues in Theory, Practice, and Policy*. Edited by Judy Illes. Oxford: Oxford University Press; 2006:245-263.

Greene J, Cohen J: **For the law, neuroscience changes nothing and everything**. In *Neuroethics: An Introduction with Readings*. Edited by Martha J. Farah. Cambridge, MA: The MIT Press; 2010:232-258.

Greenhouse CJ: **Law**. In *A Companion to Moral Anthropology.* Edited by Didier Fassin. Hoboken, New Jersey: Wiley-Blackwell; 2012:432-448.

Kolber AJ: **Free will as a matter of law**. In *Philosophical Foundations of Law and Neuroscience*. Edited by Michael S. Pardo and Dennis Patterson. Oxford: Oxford University Press; 2016:15-32.

Morse SJ: **Brain overclaim syndrome and criminal responsibility: a diagnostic note**. In *Neuroethics: An Introduction with Readings*. Edited by Martha J. Farah. Cambridge, MA: The MIT Press; 2010:268-280.

Patterson D, Pardo MS: **Philosophy, neuroscience and law: the conceptual and empirical, rule-following, interpretation and knowledge**. In *Problems of Normativity, Rules and Rule-Following. In Problems of Normativity, Rules and Rule-Following*. Edited by Michał Araszkiewicz, Paweł Banaś, Tomasz Gizbert-Studnicki, Krzysztof Płeszka. Cham, Switzerland: Springer; 2015:177-188.

Presidential Commission for the Study of Bioethical Issues. **Neuroscience and the legal system**. In their *Gray Matters: Topics at the Intersection of Neuroscience, Ethics, and Society (Vol. 2)*. Washington, DC: Presidential Commission for the Study of Bioethical Issues; 2015:85-115.

President’s Council on Bioethics Staff: **An overview of the impact of neuroscience evidence in criminal law.** In *Neuroethics: An Introduction with Readings*. Edited by Martha J. Farah. Cambridge, MA: The MIT Press; 2010:220-231.

Spranger TM: **Neurosciences and the law**. In *International Neurolaw: A Comparative Analysis*. Edited by Tade Matthias Spranger. New York: Springer-Verlag; 2012: 1-10.

Winslade WJ: **Traumatic brain injury and legal responsibility.** In *Neuroethics: Mapping the Field: Conference Proceedings*. Edited by Steven J. Marcus. New York: The Dana Press; 2002:74-82.

Wolpe PR, Foster KR, Langleben DD: **Emerging neurotechnologies for lie detection: promises and perils**. In *Neuroethics: An Introduction with Readings*. Edited by Martha J. Farah. Cambridge, Mass.: MIT Press; 2010:165-184.

## Neuroethics and policy and politics

**Articles:**

Abi-Rached JM: **The implications of the new brain sciences: the “Decade of the Brain” is over but its effects are now becoming visible as neuropolitics and neuroethics, and in the emergence of neuroeconomies**. *EMBO Rep* 2008, **9**(12):1158-1162. doi:10.1038/embor.2008.211.

Appel JM: **When the boss turns pusher: a proposal for employee protections in the age of cosmetic neurology**. *J Med Ethics* 2008, **34**(8):616-618. doi:10.1136/jme.2007.022723.

Benedikter R, Giordano J: **Neurotechnology: new frontiers for European policy.** *Pan Euro Network Sci Tech* 2012, **3**:204-207.

Bird SJ: **Potential for bias in the context of neuroethics. Commentary on "Neuroscience, neuropolitics and neuroethics: the complex case of crime, deception and fMRI"**.

*Sci Eng Ethics* 2012, **18**(3):593-600. doi:10.1007/s11948-012-9399-y.

Blank RH: **Policy implications of the new neuroscience.** *Camb Q Healthc Ethics* 2007, **16**(2): 169-180. doi:10.1017/S0963180107070193.

Cabrera LY et al.: **Brain matters: from environmental ethics to environmental neuroethics**. *Environ Health* 2016, 15:20. doi:10.1186/s12940-016-0114-3.

Caulfield T, Ogbogu U: **Biomedical research and the commercialization agenda: a review of main considerations for neuroscience**. *Account Res* 2008, **15**(4):303-320. doi:10.1080/08989620802388788.

Cohen AO, Casey BJ: **Rewiring juvenile justice: the intersection of developmental neuroscience and legal policy.** *Trends Cogn Sci* 2014, **18**(2):63-65. doi:10.1016/j.tics.2013.11.002.

Decker M, Fleischer T: **Contacting the brain--aspects of a technology assessment of neural implants**. *Biotechnol J* 2008, **3**(12):1502-1510. doi:10.1002/biot.200800225.

De Vries R: **Who will guard the guardians of neuroscience? firing the neuroethical imagination**. *EMBO Rep* 2007, **8**:S65-69. doi:10.1038/sj.embor.7401010.

Dolan TE: **Does the principle of informed consent apply to futures studies research?** *Futures* 2015, **71**:114-121. doi:10.1016/j.futures.2014.09.002.

Dubljevic V: **Toward a legitimate public policy on cognition-enhancement drugs.** *AJOB Neurosci* 2012, **3**(3):29-33. doi:10.1080/21507740.2012.700681.

Eaton ML, Illes J: **Commercializing cognitive neurotechnology--the ethical terrain**. *Nat Biotechnol* 2007, **25**(4):393-397. doi:10.1038/nbt0407-393.

Farah MJ: **Emerging ethical issues in neuroscience**. *Nat Neurosci* 2002, **5**(11):1123-1129. doi:10.1038/nn1102-1123.

Fins JJ, Shapiro ZE: **Neuroimaging and neuroethics: clinical and policy considerations.** *Curr Opin Neurol* 2007, **20**(6):650-654. doi:10.1097/WCO.0b013e3282f11f6d.

Fisher CE, Chin L, Klitzman R: **Defining neuromarketing: practices and professional challenges**. *Harv Rev Psychiatry* 2010, **18**(4):230-237. doi:10.3109/10673229.2010.496623.

Fry CL: **A descriptive social neuroethics is needed to reveal lived identities**. *Am J Bioeth* 2009, **9**(9):16-17. doi:10.1080/15265160903098580.

Fukushi T, Sakura O: **神経倫理の導入:脳卒中から、脳研究の学問領域を超えて [introduction of neuroethics: out of clinic, beyond academia in human brain research]**. *Rinsho Shinkeigaku* 2008, **48**(11):952-954.

Garnett A et al.: **Neuroethics and fMRI: mapping a fledgling relationship**. *PLoS One* 2011, **6**(4):e18537. doi:10.1371/journal.pone.0018537.

Giordano J: **Neuroethical issues in neurogenetics and neurotransplantation technology – the need for pragmatism and preparedness in practice and policy.** *Studies Ethics, Law Technol* 2011,**4**(3):4. doi:10.2202/1941-6008.1152.

Giordano J, Forsythe C, Olds J. **Neuroscience, neurotechnology and national security: the need for preparedness and an ethics of responsible action**. *AJOB Neurosci* 2010, **1**(2):35-36. doi:10.1080/21507741003699397.

Giordano J, DuRousseau, D: **Toward right and good use of brain-machine interfacing neurotechnologies: ethical issues and implications for guidelines and policy.** *Cog Technol* 2010, **15**(2):5-10.

Hall ZW: **Mapping the future**. *Cerebrum* 2002, **4**(3):72-76.

Hoffman MB: **Neuroscience cannot answer these questions: a response to G. and R. Murrow's essay hypothesizing a link between dehumanization, human rights abuses and public policy.** *J Law Biosci* 2015, **3**(1):167-173. doi:10.1093/jlb/lsv041.

Hutchinson P: **Third International Satellite Conference on Neuro-Chemical Monitoring: proposed guidelines for neuro-chemical monitoring**. *Acta Neurochir Suppl* 2002, **81**:341-342. doi:10.1007/978-3-7091-6738-0_86.

Illes J, Pierce R: **Introduction: accountability in neuroethics**. *Account Res* 2008, **15**(4):205-208. doi:10.1080/08989620802388648.

Kagawa C: **神経倫理と生命倫理 - バルカン化論争の意味 [neuroethics and bioethics - implications of Balkanization controversy]**. *Brain Nerve* 2009, **61**(1):11-17.

Kang SM, Inzlicht M, Derks B: **Social neuroscience and public policy on intergroup relations: a Hegelian analysis.** *J Soc Issues* 2010, **66**(3): 585-601. doi:10.1111/j.1540-4560.2010.01664.x.

Katayama Y, Fukaya C: **深い脳の刺激と神経倫理 [deep brain stimulation and neuroethics]**. *Brain Nerve* 2009, **61**(1):27-32.

Keel BA: **Protecting America’s secrets while maintaining academic freedom**. *Acad Med* 2004, **79**(4):333-342.

Kenning PH, Linzmajer M: **Consumer neuroscience: an overview of an emerging discipline with implications for consumer policy.** *J Verbrauch Lebensm* 2011, **6**(1):111-125. doi: 10.1007/s00003-010-0652-5.

Koenig BA, et al.: **Brain science and social policy**. *Cerebrum* 2002, **4**(3):59-62.

Lee N, Broderick AJ, Chamberlain L: **What is “neuromarketing”? a discussion and agenda for future research**. *Int J Psychophysiol* 2007, **63**(2):199-204. doi:10.1016/j.ijpsycho.2006.03.007.

Lyng S: **Brain, body, and society: bioethical reflections on socio-historical neuroscience and neuro-corporeal social science**. *Am J Bioeth* 2009, **9**(9):25-26. doi:10.1080/15265160903095982.

Martin M, Fangerau H, Karenberg A: **German neurology and the ‘Third Reich’**. *Eur Neurol* 2016, **76**(5-6):234-243. doi:10.1159/000450851.

Millei Z, Joronen M: **The (bio)politicization of neuroscience in Australian early years policies: fostering brain-resources as human capital**. *J Educ Policy* 2016, **31**(4):389-404. doi:10.1080/02680939.2016.1148780.

Mima T: **最近の神経科学の進歩による社会的影響 [social impact of recent advances in neuroscience]**. *Brain Nerve* 2009, **61**(1):18-26.

Moreno JD: **DARPA on your mind**. *Cerebrum* 2004, **6**(4):91-99.

Moreno JD: **Society and the reception of imaging technology: the American experience**. *Cortex* 2011, **47**(10):1256-1258. doi:10.1016/j.cortex.2011.04.024.

Munro GD, Munro CA: **“Soft” versus “hard” psychological science: biased evaluations of scientific evidence threatens or supports a strongly held political identity.** *Basic Appl Soc Psych* 2014, **36**(6):533-543. doi:10.1080/01973533.2014.960080.

Murrow GB, Murrow R: **A hypothetical neurological association between dehumanization and human rights abuses.** *J Law Biosci* 2015, **2**(2):336-364. doi:10.1093/jlb/lsv015.

O’Connell G: **Tracking the impact of neuroethics**. *Cortex* 2011, **47**(10):1259-1260. doi:10.1016/j.cortex.2011.04.020.

O’Mara S: **Torturing the brain: on the folk psychology and folk neurobiology motivating “enhanced and coercive interrogation techniques”**. *Trends Cogn Sci* 2009, **13**(12):497-500. doi:10.1016/j.tics.2009.09.001.

Paylor B, Longstaff H, Rossi F, Illes J: **Collision or convergence? beliefs and politics in neuroscience discovery, ethics, and intervention.** *Trends Neurosci* 2014, **37**(8):409-412. doi:10.1016/j.tins.2014.06.001.

Plischke H, DuRousseau D, Giordano, J: **EEG-based neurofeedback: the promise of neurotechnology and need for neuroethically-informed guidelines and policies**. *Ethics Biol Eng Med* 2011, **2**(3):221-232. doi:10.1615/EthicsBiologyEngMed.2012004853.

Pustovrh T, Mali F: **Exploring some challenges of the pharmaceutical cognitive enhancement discourse: users and policy recommendations**. *Neuroethics* 2014, **7**(2):137-158. doi:10.1007/s12152-013-9192-x.

Seymour B, Vlaev I: **Can, and should, behavioural neuroscience influence public policy?** *Trends Cogn Sci* 2012, **16**(9):449-451. doi:10.1016/j.tics.2012.07.005.

Shook JR, Galvagni L, Giordano J: **Cognitive enhancement kept within contexts: neuroethics and informed public policy.** *Front Syst Neurosci* 2014, **8**:228. doi:10.3389/fnsys.2014.00228.

Taylor PL: **Responsibility rewarded: ethics, engagement, and scientific autonomy in the labyrinth of the minotaur**. *Neuron* 2011, **70**(4):577-581. doi:10.1016/j.neuron.2011.05.009.

Tennison MN, Moreno JD: **Neuroscience, ethics, and national security: the state of the art**. *PLoS Biol* 2012, **10**(3):e1001289. doi:10.1371/journal.pbio.1001289.

Timpane J: **Models for the neuroethical debate in the community**. *Cerebrum* 2004, **6**(4):100-107.

Vrecko S: **Neuroscience, power and culture: an introduction**. *Hist Human Sci* 2010, **23**(1):1-10. doi:10.1177/0952695109354395.

Wolpe PR: **Ethics and social policy in research on the neuroscience of human sexuality**. *Nat Neurosci* 2004, **7**(10):1031-1033. doi:10.1038/nn1324.

Zimmerman E, Racine E: **Ethical issues in the translation of social neuroscience: a policy analysis of current guidelines for public dialogue in human research**. *Account Res* 2012, **19**(1):27-46. doi:10.1080/08989621.2012.650949.

**Books**

Blank RH: *Intervention in the Brain: Politics, Policy, and Ethics*. Cambridge, Mass.: MIT Press; 2013.

Jotterand J, Dubljević V: *Cognitive Enhancement: Ethical and Policy Implications in International Perspectives*. Oxford: Oxford University Press; 2016.

Illes J: *Neuroethics: Defining the Issues in Theory, Practice, and Policy*. Oxford: Oxford University Press; 2006.

Marcus GE: *Political Psychology: Neuroscience, Genetics, and Politics* **.** New York: Oxford University Press; 2013.

Pitts-Taylor V: *The Brain’s Body: Neuroscience and Corporeal Politics*. Durham: Duke University Press; 2016.

Pykett J: *Brain Culture: Shaping Policy Through Neuroscience*. Chicago, IL: Policy Press; 2015.

**Book Chapters**

Blank RH: **Framing cognitive enhancement policy**. In his *Cognitive Enhancement: Social and Public Policy Issues*. New York: Palgrave Pivot; 2015:92-108.

Blank RH: **Policy and politics of cognitive enhancement**. In his *Cognitive Enhancement: Social and Public Policy Issues*. New York: Palgrave Pivot; 2015:71-91.

Blank RH: **Regulating cognitive enhancement technologies: policy options and problems.** In *Cognitive Enhancement: Ethical and Policy Implications in International Perspectives.* Edited by Fabrice Jotterand and Veljko Dubljević. New York: Oxford University Press; 2016:239-258.

Boire RG: **State-imposed brain intervention: the case of pharmacotherapy for drug abuse.** In *Neuroethics: An Introduction with Readings*. Edited by Martha J. Farah. Cambridge, MA: MIT Press; 2010:281-294.

Bostrom N, Sandberg A: **Cognitive enhancement: methods, ethics, regulatory challenges**. In *Contemporary Issues in Bioethics*. 8th edition. Edited by Tom L. Beauchamp, LeRoy Walters, Jeffrey P. Kahn, Anna C. Mastroianni. Boston: Wadsworth; 2014:579-588.

Brown TR, McCormick JB: **New directions in neuroscience policy**. In *Oxford Handbook of Neuroethics*. Edited by Judy Illes and Barbara J. Sahakian, J. Oxford: Oxford University Press; 2011:675-700.

Casebeer WD: **Postscript: a neuroscience and national security normative framework for the twenty-first century**. In *Neurotechnology in National Security and Defense: Practical Considerations, Neuroethical Concerns*. Edited by James Giordano. Boca Raton: CRC Press; 2015:279-283.

Greely HT et al.: **Toward responsible use of cognitive-enhancing drugs by the healthy: policy suggestions**. In *Neuroethics: An Introduction with Readings*. Edited by Martha J. Farah. Cambridge, Mass.: MIT Press; 2010:73-77.

Murphy N: **From neurons to politics – without a soul.** In *Neuroethics: An Introduction with Readings*. Edited by Martha J. Farah. Cambridge, Mass.: MIT Press; 2010:357-363.

Ng E: **The critique of mindfulness and the mindfulness of critique: paying attention to the politics of ourselves with Foucault’s analytic of governmentality**. In *Handbook of Mindfulness: Culture, Context, and Social Engagement*. Edited by Ronald E. Purser, David Forbes, T. Adam Burke. Cham, Switzerland: Springer International Publishing; 2016:135-152.

Sarewitz D, Karas TH: **Policy implications of technologies for cognitive enhancement**. In *Neurotechnology: Premises, Potential, and Problems*. Edited by James Giordano. Boca Raton: CRC Press; 2012:267-285.

Tabery J: **Can (and should) we regulate neurosecurity? lessons from history**. In *Neurotechnology in National Security and Defense: Practical Considerations, Neuroethical Concerns*. Edited by James Giordano. Boca Raton: CRC Press; 2015:249-258.

Tovino S: **Regulating neuroimaging.** In *Neuroethics: An Introduction with Readings*. Edited by Martha J. Farah. Cambridge, Mass.: MIT Press; 2010:201-210.

Tractenberg RE, FitzGerald KT, Giordano J: **Engaging neuroethical issues generated by the use of neurotechnology in national security and defense: toward process, methods, and paradigm**. In *Neurotechnology in National Security and Defense: Practical Considerations, Neuroethical Concerns*. Edited by James Giordano. Boca Raton: CRC Press; 2015:259-277.

## International neuroethics

**Articles**

Baharuddin A, Musa MN, Salleh SS: **A preliminary insight into an Islamic mechanism for neuroethics**. *Malays J Med Sci* 2016, **23**(1):1-3.

Benedikter R, Giordano J: **The outer and inner transformation of the global sphere through technology: the state of two fields in transition**. *New Global Studies* 2011, **5**(2). doi:10.2202/1940-0004.1129.

Brindley T, Giordano J: **International standards for intellectual property protection of neuroscience and neurotechnology: neuroethical, legal and social (NELS) considerations in light of globalization**. *Stanford J Law Sci Policy* 2014, **7**:33-49.

Christen M et al.: **Ethical challenges of simulation-driven Big Neuroscience**. *AJOB Neurosci* 2016, **7**(1):5-17. doi:10.1080/21507740.2015.1135831.

Cotter LB et al.: **Building global capacity for brain and nervous system disorders research**. *Nature* 2015, **527**(7578):S207-S213. doi:10.1038/nature16037.

Eguchi S: **Conflict of interest regarding clinical psychiatrist relationship with global pharmaceutical industries**. *Seishin Shinkeigaku Zasshi* 2010, **112**(11):1117-1123.

Figueroa G: **Neuroethics: the pursuit of transforming medical ethics in scientific ethics**. *Biol Res* 2016, **49**:11. doi:10.1186/s40659-016-0070-y.

Fukushi T, Sakura O: **日本における神経倫理 - 現状と今後のビジョン [neuroethics in Japan - current view and future visions]**. *Brain Nerve* 2009, **61**(1):5-10.

Giordano J, Alam S: **Ethical considerations in the globalization of medicine - an interview with Dr. James Giordano**. *BMC Med* 2013, **11**:69. doi:10.1186/1741-7015-11-69.

Giordano J, Lanzilao E, Shook JR, Benedikter R: **Guidare la neuroscienza e lo sviluppo della persona nel XXI secolo: una prospettiva naturalistica e cosmopolita per la neuroetica [driving neuroscience and personal development in the 21st century: a naturalistic and cosmopolitan perspective for neuroethics**. *L’Arco di Giano* 2015, 80:147-164.

Greely HT, Ramos KM, Grady C: **Neuroethics in the age of Brain Projects**. *Neuron* 2016, **92**(3):637-641. doi:10.1016/j.neuron.2016.10.048.

Illes J et al.: **International perspectives on engaging the public in neuroethics**. *Nat Rev Neurosci* 2005, **6**(12):977-982. doi:10.1038/nrn1808.

Kalichman M, Plemmons D, Bird SJ: **Editors’ overview: neuroethics: many voices and many stories**. *Sci Eng Ethics* 2012, **18**(3):423-432. doi:10.1007/s11948-012-9398-z.

Lanzilao E, Shook JR, Newman R, Giordano J. **Advancing neuroscience on the 21st century world stage: the need for - and proposed structure of - an internationally relevant neuroethics**. *Ethics Biol Engineer Med* 2013, **4**(3):211-229. doi:10.1615/EthicsBiologyEngMed.2014010710.

Leefmann J, Levallois C, Hildt E: **Neuroethics 1995-2012: a bibliometric analysis of the guiding themes of an emerging research field**. *Front Hum Neurosci* 2016, **10**:336. doi:10.3389/fnhum.2016.00336.

Lombera S, Illes J: **The international dimensions of neuroethics**. *Dev World Bioeth* 2009, **9**(2):57-64. doi:10.1111/j.1471-8847.2008.00235.x.

Shook JR, Giordano J: **A principled, cosmopolitan neuroethics: considerations for international relevance**. *Philos Ethics Humanit Med* 2014, **9**:1. doi:10.1186/1747-5341-9-1.

Stein DJ, Giordano J. **Global mental health and neuroethics**. *BMC Med* 2015, **13**:44. doi:10.1186/s12916-015-0274-y.

Strous RD, Jotkowitz A: **Ethics and research in the service of asylum seekers**. *Am J Bioeth* 2010, **10**(2):63-65. doi:10.1080/15265160903506400.

Toope SJ: **Internationalism and global norms for neuroethics**. *Am J Bioeth* 2009, **9**(1):1-2. doi:10.1080/15265160802617902.

Tucker JB: **Preventing the misuse of biology: lessons from the oversight of smallpox virus research**. *Int Secur* 2006, **31**(2):116-150. doi:10.1162/isec.2006.31.2.116.

**Books**

Jotterand F, Dubljevic V: *Cognitive Enhancement: Ethical and Policy Implications in International Perspectives*. New York: Oxford University Press; 2016.

Spranger TM: *International Neurolaw: A Comparative Analysis*. Heidelberg: Springer; 2012.

Terbeck S: *The Social Neuroscience of Intergroup Relations: Prejudice, Can We Cure It?* New York: Springer; 2016.

Warnick JE, Landis D: *Neuroscience in Intercultural Contexts*. New York: Springer; 2015.

**Book Chapters**

Anderson MA, Fitz, N, Howlader D: **Neurotechnology research and the world stage: ethics, biopower, and policy**. In *Neurotechnology: Premises, Potential, and Problems*. Edited by James Giordano. Boca Raton: CRC Press; 2012:287-300.

Blank RH: **Globalization--pluralist concerns and contexts: shaping international policy in neuroethics**. In *Scientific and Philosophical Perspectives in Neuroethics*. Edited by James Giordano, Bert Gordijn. Cambridge: Cambridge University Press; 2010:321-342.

Brown RE: **Seven steps to setting up a neuroscience program in a developing country**. In *Neuroscience in the 21st Century*. Edited by Donald W. Pfaff, Nora D. Volkow. New York: Springer; 2016:4087-4114.

Chen D, Quirion R: **From the internationalization to the globalization of neuroethics: some perspectives and challenges**. In *Oxford Handbook of Neuroethic*s. Edited by Judy Illes and Barbara J. Sahakian. Oxford: Oxford University Press; 2011:823-834.

Evert J, Huish R, Heit G: **Global health ethics**. In *Oxford Handbook of Neuroethic*s. Edited by Judy Illes and Barbara J. Sahakian. Oxford: Oxford University Press; 2011:835-855.

Giordano J: **Neurotechnology**. In *Encyclopedia of Global Bioethics*. Edited by Henk ten Have. Dordrecht: Springer International Publishing; 2015:1-8

Giordano J, Benedikter R: **Neurotechnology, culture, and the need for a cosmopolitan neuroethics**. In *Neurotechnology: Premises, Potential, and Problems*. Edited by James Giordano. Boca Raton: CRC Press; 2012:233-241.

Giordano J: **Neurotechnology, global relations, and national security: shifting contexts and neuroethical demands**. In his *Neurotechnology in National Security and Defense: Practical Considerations, Neuroethical Concerns*. Boca Raton: CRC Press; 2015:1-10.

Glannon W: **Neuroethics**. In *Encyclopedia of Global Bioethics*. Edited by Henk ten Have. Dordrecht: Springer International Publishing; 2015:1-12.

Novossiolova T: **Biosecurity as a normative challenge**. In *Handbook of Neuroethics*. Edited by Jens Clausen, Neil Levy. Dordrecht: Springer; 2015:1813-1825.

Pykett J: **From global economic change to neuromolecular capitalism**. In *Neuroscience and Critique: Exploring the Limits of the Neurological Turn*. Edited by Jan De Vos, Ed Pluth. New York: Routledge; 2016:81-99.

Reichlin M: **Neuroethics**. In *Companion to Moral Anthropology*. Edited by Didier Fassin. Hoboken: Wiley Blackwell; 2015:595-610.

## “Trans”/“post” human issues

**Articles**

Agar N: **Whereto transhumanism? the literature reaches a critical mass**. *Hastings Cent Rep* 2007, **37**(3):12-17. doi:10.1353/hcr.2007.0034.

Anderson M, Ishiguro M, Fukushi T: **“Involving interface”: an extended mind theoretical approach to roboethics**. *Account Res* 2010, **17**(6):316-329. doi:10.1080/08989621.2010.524082.

Attiah MA, Farah MJ: **Minds, motherboards, and money: futurism and realism in the neuroethics of BCI technologies**. *Front Syst Neurosci* 2014, **8**:86. doi:10.3389/fnsys.2014.00086.

Béland JP et al.: **The social and ethical acceptability of NBICs for purposes of human enhancement: why does the debate remain mired in impasse?** *Nanoethics* 2011, **5**(3):295-307. doi:10.1007/s11569-011-0133-z.

Benanti P: **Neuroenhancement in young people: cultural need or medical technology?** *AJOB Neurosci* 2010, **1**(1):27-29. doi:10.1080/21507740903523210.

Benedikter R, Giordano J, FitzGerald K: **The future of the self-image of the human being in the age of transhumanism, neurotechnology and global transition**. *Futures* 2010, **42**(10):1102–9. doi:10.1016/j.futures.2010.08.010.

Bess M: **Enhanced humans versus "normal people": elusive definitions.** *J Med Philos* 2010, **35**(6):641-655. doi:10.1093/jmp/jhq053.

Bostrom N: **In defense of posthuman dignity.** *Bioethics* 2005, **19**(3):202-214. doi:10.1111/j.1467-8519.2005.00437.x.

Bredenoord AL, van der Graaf R, van Delden JJ: **Toward a "post-posthuman dignity area" in evaluating emerging enhancement technologies.** *Am J Bioeth* 2010, **10**(7):55-57. doi:10.1080/15265161003686514.

Bunting M: **There is no stop button…** *Ecologist* 2006, **36**(2):16-17.

Cabrera Trujillo LY, Engel-Glatter S: **Human-animal chimera: a neuro driven discussion? comparison of three leading European research countries**. *Sci Eng Ethics* 2015, **21**(3):595-617. doi:10.1007/s11948-014-9556-6.

Chapman AR: **Inconsistency of human rights approaches to human dignity with transhumanism.** Am J Bioeth. 2010, **10**(7):61-63. doi:10.1080/15265161003702899.

Elliott C: **Humanity 2.0.** *Wilson Q* 2003, **27**(4):13-20.

Farah MJ: **An ethics toolbox for neurotechnology.** *Neuron* 2015, **86**(1):34-37. doi:10.1016/j.neuron.2015.03.038.

Frippiat L: **[Human technical enhancement: an introduction to the currents of the debate as well as their fracture lines].** *J Int Bioethique* 2011, **22**(3-4):33-50, 192.

Galagan P: **How would you train a transhuman?** *Training and Development* 2012, **1**:27-29.

Glenn LM, Dvorsky G: **Dignity and agential realism: human, posthuman, and nonhuman.** *Am J Bioeth* 2010, **10**(7):57-58. doi:10.1080/15265161003686548.

Goffi JY: **[Human nature and the enhancement of human beings in the light of the transhumanist program].** *J Int Bioethique* 2011, **22**(3-4):19-32, 191.

Harris LJ, Almerigi JB: **Probing the human brain with stimulating electrodes: the story of Roberts Bartholow’s (1874) experiment on Mary Rafferty**. *Brain Cogn* 2009, **70**(1):92-115. doi:10.1016/j.bandc.2009.01.008.

Horgan J: **The forgotten era of brain chips**. *Sci Am* 2005, **293**(4):66-73. doi:10.1038/scientificamerican1005-66.

Hottois G: **Is transhumanism a humanism?** *Rev Derecho Genoma Hum* 2015, **42**:15-24.

Hughes J: **Contradictions from the Enlightenment roots of transhumanism.** *J Med Philos* 2010, **35**(6):622-640. doi:10.1093/jmp/jhq049.

Jotterand F: **Human dignity and transhumanism: do anthro-technological devices have moral status?** *Am J Bioeth* 2010, **10**(7):45-52. doi: 10.1080/15265161003728795.

Koch T: **Enhancing who? enhancing what? ethics, bioethics, and transhumanism.** J Med Philos 2010, **35**(6):685-699. doi:10.1093/jmp/jhq051.

Lee J: **Cochlear implantations, enhancements, transhumanism and posthumanism: some human questions**. *Sci Eng Ethics* 2016, **22**(1):67-92. doi:10.1007/s11948-015-9640-6.

Maestrutti M: **Cyborg identities and contemporary techno-utopias: adaptations and transformations of the body in the age of nanotechnology.** *J Int Bioethique* 2011, **22**(1):71-88, 209-10.

Mavridou O: **The monstrous transformation of the self: translating Japanese cyberpunk and the posthuman into the living world**. *Luminary* 2015, **6**(1):71-86.

McGee EM, Maguire GQ: **Becoming borg to become immortal: regulating brain implant technologies**. *Camb Q Healthc Ethics* 2007, **16**(3):291-302. doi:10.1017/S0963180107070326.

McNamee MJ, Edwards SD:. **Transhumanism, medical technology and slippery slopes.** *J Med Ethics* 2006, **32**(9):513-518. doi:10.1136/jme.2005.013789.

Mičunović M, Badurina B, Bosančić B: **Pojav tehnoloske triade: opisni pojem danasnje totalne realnosti [The occurrence of technological triad: descriptive concept of today's totality of reality]**. *Knjiznica*2016, **60**(1):135-160.

Murillo JI: **Does post-humanism still need ethics? the normativity of an open nature.** *Cuad Bioet* 2014, **25**(85):469-479.

Nouvel P: [**From synthetic biology to synthetic humankind**]. *C R Biol* 2015, **338**(8-9):559-565. doi:10.1016/j.crvi.2015.06.015.

Pastor LM, Garcia Cuadrado JÁ: **Modernity and postmodernity in the genesis of transhumanism-posthumanism.** *Cuad Bioét* 2014, **25**(85):335-350.

Podroužková J: **Personal identity in enhancement.** *Ostium* 2015, **11**(3):1-7.

Proust J: **Cognitive enhancement, human evolution and bioethics.** *J Int Bioethique* 2011, **22**(3-4):153-173, 199.

Rodriguez PE: **¿Tiene sentido hablar de poshumanismo? acerca de la relación entre teoría de la comunicación y biopolítica de la información [Does it make sense to talk about posthumanism? About the relationship between communication theory and biopolitics of information]**. Galáxia 2010, 9-12.

Sadler JZ: **Dignity, arête, and hubris in the transhumanist debate.** *Am J Bioeth* 2010, **10**(7):67-68. doi:10.1080/15265161003728845.

Shatzer J: **A posthuman liturgy? virtual worlds, robotics, and human flourishing.** *New Bioeth* 2013, **19**(1):46-53.

Shook JR, Giordano J: **Neuroethics beyond normal: performance enablement and self-transformative technologies.** *Camb Q Healthc Ethics* 2016, **25**(1):121-140. doi:10.1017/S0963180115000377.

Stile GC: **Transumanesimo: una introduzione all’idea di evoluzione autodiretta [Transhumanism: an introduction to the idea of self-directed evolution]**. *ISPF-LAB* 2015, **12**:1-12. doi:10.12862/ISPF15L406.

Sutton A: **Transhumanism: a new kind of Promethean hubris.** *New Bioeth* 2015, **21**(2):117-27. doi:10.1179/2050287715Z.00000000060.

Tennison MN: **Moral transhumanism: the next step**. *J Med Philos* 2012, **37**(4):405-416. doi: 10.1093/jmp/jhs024.

Valera L: **Posthumanism: beyond humanism?** *Cuad Bioét* 2014, **25**(3):481-491.

Van Hilvoorde I, Landeweerd L: **Enhancing disabilities: transhumanism under the veil of inclusion?** *Disabil Rehabil* 2010, **32**(26):2222-2227. doi: 10.3109/09638288.2010.491578.

Woll S: **Transhumanismus und posthumanismus – ein überblick oder: der schmale grat zwischen utopie und dystopie [transhumanism and posthumanism – an overview: the narrow ridge between utopia and dystopia]**. *J New Front Spat Con* 2013, **5**:43-48.

Zehr EP: **Future think: cautiously optimistic about brain augmentation using tissue engineering and machine interface**. *Front Syst Neurosci* 2015, **72**. doi:10.3389/fnsys.2015.00072.

Zehr EP: **The potential transformation of our species by neural enhancement.** *J Mot Behav* 2015, **47**(1):73-78. doi:10.1080/00222895.2014.916652.

**Books**

Bostrom N: *Superintelligence: Paths, Dangers, Strategies*. Oxford: Oxford University Press; 2016.

Braidotti R: *The Posthuman*. Cambridge, UK: Polity Press; 2013.

Campa R: *Humans and Automata: A Social Study of Robotics*. Frankfurt: Peter Lang; 2015.

Cole-Turner R: *Transhumanism and Transcendence: Christian Hope in an Age of Technological Enhancement*. Washington, DC: Georgetown University Press; 21011

Cooter R, Stein C: *Writing History in the Age of Biomedicine*. New Haven: Yale University Press; 2013.

Fukuyama F: *Our Posthuman Future: Consequences of the Biotechnology Revolution*. New York: Farrar, Straus and Giroux; 2002.

Fuller S, Lipińska V: *The Proactionary Imperative: A Foundation for Transhumanism*. Basingstoke, UK: Palgrave Macmillan; 2014.

Giordano J: *Neurotechnology: Premises, Potential, and Problems.* Boca Raton: CRC Press; 2012.

Gordijn B, Chadwick RF: *Medical Enhancement and Posthumanity*. Dordrecht: Springer; 2008.

Grebowicz M, Merrick H: *Beyond the Cyborg: Adventures with Donna Haraway*. New York: Columbia University Press; 2013.

Haraway DJ: *The Haraway Reader*. New York: Routledge; 2004.

Haraway DJ: *Manifestly Haraway*. Minneapolis, MN: University of Minnesota Press; 2016.

Haraway DJ: *Simians, Cyborgs, and Women: The Reinvention of Nature*. New York: Routledge; 2015.

Herold E: Beyond Human: *How Cutting-Edge Science Is Extending Our Lives*. New York: St. Martin’s Press; 2016.

Lake CB: *Prophets of the Posthuman: American Fiction, Biotechnology, and the Ethics of Personhood*. Notre Dame, IN: University of Notre Dame Press; 2013.

Le Dévédec N: *La Société de l'Amélioration: La Perfectibilité Humaine des Lumières au Transhumanisme [The Society of Improvement: Human Perfectibility from the Enlightenment to Transhumanism]*. Montréal: Liber; 2015.

Linke DB: *Die Dritte Natur: Über Posthumane Faktizität [The Third Nature: About Posthuman Facts]*. Munster: Lit; 2004.

Mercer C, Trothen TJ: *Religion and Transhumanism: The Unknown Future of Human Enhancement*. Santa Barbara, CA: Praeger; 2015.

Nayar PK: *Posthumanism*. Cambridge: Polity; 2014.

Roden D: *Posthuman Life: Philosophy at the Edge of the Human*. London: Routledge; 2015.

Sharon T: *Human Nature In An Age of Biotechnology: The Case for Mediated Posthumanism*. Dordrecht: Springer; 2014.

Sorgner SL: *Transhumanismus: "Die Gefährlichste Idee der Welt"!? [Transhumanism: “The Most Dangerous Idea in the World!?”]*. Freiburg: Herder; 2016.

Tirosh-Samuelson H, Mossman KL: *Building Better Humans? Refocusing the Debate on Transhumanism*. Frankfurt: Peter Lang; 2012.

Young S: *Designer Evolution: A Transhumanist Manifesto*. Amherst, NY: Prometheus Books; 2006.

**Book Chapters**

Benanti P: **The cyborg and cyborgization**. In *Neurotechnology: Premises, Potential, and Problems*. Edited by James Giordano. Boca Raton: CRC Press; 2012:191-198.

Fryer DR: **On the possibilities of posthumanism, or how to think queerly in an anti-black world**. In his *Thinking Queerly: Race, Class, Gender, and the Ethics of Identity*. Boulder, CO: Paradigm Publishers; 2012:1-12.

Hall JS: **Improvements: a perspective on transhumanism**. In his *Nanofuture: What’s Next for Nanotechnology*. Amherst, NY: Prometheus Books; 2005:257-270.

Herold E: **Just don’t call it transhumanism**. In her *Beyond Human: How Cutting-Edge Science Is Extending Our Lives*. New York: St. Martin’s Press; 2016:238-263.

Hook CC: **Techno sapien: nanotechnology cybernetics, transhumanism and the remaking of humankind**. In: *Human Dignity in the Biotech Century: A Christian Vision for Public Policy*. Edited by Charles W. Colson and Nigel M. de S. Cameron. Downers Grove, IL: InterVarsity Press; 2004:75-97.

Hook CC: **Transhumanism and posthumanism**. In *Encyclopedia of Bioethics*, 3rd ed. Edited by Stephen G. Post. New York: Macmillan Reference USA; 2004:2517-2520.

Hottois G, Missa JN, Perbal L: **Technoscience et médecine d'amélioration**. In *L'humain et Ses Préfixes: Une Encyclopédie du Transhumanisme et du Posthumanisme*. Edited by Gilbert Hottois, Jean-Noël Missa, Laurence Perbal. Paris: J. Vrin; 2015:193-342.

McKenny GP: **The ethics of regenerative medicine: beyond humanism and posthumanism**. In *Contemporary Issues in Bioethics*. 8th edition. Edited by Tom L. Beauchamp, LeRoy Walters, Jeffrey P. Kahn, Anna C. Mastroianni. Boston: Wadsworth; 2014:588-595.

Schneider S: **Future minds: transhumanism, cognitive enhancement, and the nature of persons**. In *The Penn Center Guide to Bioethics*. Edited by Vardit Ravitsky, Autumn Fiester, Arthur L. Caplan. New York: Springer; 2009:95-110.

Sheehan P: **Posthuman bodies**. In *The Cambridge Companion to the Body in Literature*. Edited by David Hillman, Ulrika Maude. New York: Cambridge University Press; 2015:245-260.

Winner L: **Resistance is futile: the posthuman condition and its advocates**. In I*s Human Nature Obsolete? Genetics, Bioengineering, and the Future of the Human Condition*. Edited by Harold W. Baillie, Timothy K. Casey. Cambridge, Mass.: MIT Press; 2005:385-411.

Wolpe PR: **Neurotechnology, cyborgs, and the sense of self**. In *Neuroethics: Mapping the Field*. Edited by Steven J. Marcus. New York: Dana Press; 2002:159-191.

## Discussion and conclusions

It is our intent that this bibliography will provide background resources that can foster a greater understanding of recent ethical issues in translational neurosciences, facilitate deeper discourse about such issues, and contribute to a more complete view of the literature in neuroethics, if not the field, at large. In regarding this work, it becomes clear that neuroethics is a sub-field of bioethics, and thus as a focused aspect of ethics in general [8–10]. The “substrates” of such ethical address and analyses are research and applications of brain science, and both are involved, to some extent, with a contingent understanding of the relationships of brain structure to functions of thought, emotion and behavior. Thus, it could be argued that many (if not all) of the questions of this field are either directly or indirectly entwined with what Chalmers has called the “hard problem” of neuroscience [11]. Does this make neuroethics somewhat different from other domains of ethical analysis? Perhaps, in that there are persistent unknowns that pervade attempts at neurological and psychiatric assessments and interventions, and the meanings and values that are derived from neuroscientific information [12, 13]. Neuroethics may not be the only field that encounters and deals with these types of ambiguities; ethical discourse about research and applications of genetics and nanoscience and technology are also plagued by such questions and issues.

But the philosophical and practical implications of the relationship of the brain to the mind (and whatever it is construed to be), self, and ontology may also raise other questions about what neuroethics is, and what it is posturing to achieve [14]. Review of the literature presented in the four parts of this bibliography will reveal that neuroethics is not epistemologically or ideologically unified in terms of considering the relationship of brain to mental functions and experiences. Studies in neuroethics tend, at the very least, to bracket reductionist views as a parochial perspective, or at most assume a deep intimacy between mental processes and the brain as an organ embodied in socially embedded – and responsive - individuals. Axiomatically, as a domain of ethics, neuroethics deals with exploring, defining and advancing the “good” of neuroscientific research and its uses, and as such cannot ignore or negate sociology, anthropology, psychology and/or human self-understanding [15, 16].

Indeed, as a discipline, and in its practice(s), neuroethics can remain on firmer scientific ground by taking into consideration mind-brain relations where they are empirically confirmed. The current stance is that this confirming evidence is not derived from the use of a single tool, such as the newest techniques of brain imaging or stimulation; and neuroethical discourse has much to do with establishing that stance. The body of neuroethical literature has included much work about the tensions between oppositional positions regarding the validity and value of certain neurological diagnostics and the relevance of those diagnostics for disputing folk psychology. Critically detached perspectives on neuroscience are not scarce. That same literature is also not lacking in scrutiny from ethicists or social scientists.

Granting all this, philosophical discussions in neuroethics have, and can still become somewhat preoccupied with the pros and cons of reductionisms of many sorts, but this is – and arguably should be – recognized as just that: philosophical discourse that offers speculations about the aforementioned “hard questions and problems,” and what these infer for views, validity, and uses of neuroscientific information and tools. In aspiring to establish neuroethics both as a sub-discipline of bioethics and ethics at large, it becomes important to demarcate its subject matter(s) and methods of inquiry. There is an adage that “…if you wish to know what something *is*, look closely at what it *does*”. So, some fifteen years after its nominal establishment, we may now look to the literature to assess what neuroethics has done, and in this way, infer what it is, and perhaps what it may become (or need to become). In this approach, we might also ask if the speculative aspects of neuroethical discourse (i.e. - in proposing what brain science might incur for the conduct of medicine and various realms of social activity) create something of a performative disposition (both for the field, and its practitioners). Surely, there are some - both within the field and external to it – who have made claims about the future impact of brain science on various dimensions of human existence (and admittedly, we have speculated upon such trajectories, as well) [17, 18]. But there have been, and continue to be calls within the neuroethical literature to avoid exaggerative assertions, and instead to focus upon clear and present questions, issues and problems [19, 20]. This does not compel completely dismissing the discipline of neuroethics, or some of its scholars for posing concerns about envisioned directions and effects of brain science. Indubitably, some presumed problems will likely dissipate for lack of scientific warrant or social relevance, while other problems will consolidate into definitive issues of looming importance that demand attention (and in the event, current speculation about such future trajectories may enable early address and intervention).

As a discipline that is focused upon science and its conduct and applications in society, neuroethics must define itself in terms of such real problems and appropriate methods. Given that both science and society change, and often in reciprocity as a consequence of their interaction, it therefore remains open, if not necessary, for neuroethics to continually reconstruct itself. The literature will be the vector and testimonial to this ongoing review and revision. Through such evolving discourse, neuroethics may address, critique and guide the brain sciences, and equally examine, critically evaluate and revise its own stances and practices. It may be, of course, that neuroethics will fail to fully achieve the status of a discrete discipline, and instead, will “revert” to being the work of a group of scholars who focus upon ethical issues in brain research, clinical neurology and psychiatry, and the employment of neuroscientific techniques in the social sphere. Or, it may be that the field’s agendas may be better handled jointly by other, established disciplines. Multi-disciplinary fields are far more numerous than unified disciplines for good reasons. So while it is certainly possible that the genuinely practical problems of brain science in society may not suffice to maintain the need for neuroethics as a discrete multidisciplinary field, we posit that they most probably will. Our hope is that this bibliography will provide a basis to both look back upon and assess the foci, scope and efforts of neuroethical discourse to date, and from this vantage point develop and enhance the discipline and practices of neuroethics and its value to brain science and society both at present, and in the future.
