# Erratum to: A four-part working bibliography of neuroethics: Part 4 - Ethical issues in clinical and social applications of neuroscience

**DOI:** 10.1186/s13010-017-0044-x

**Published:** 2017-06-28

**Authors:** Kira Becker, John R. Shook, Martina Darragh, James Giordano

**Affiliations:** 1 Department of Neuroscience, Amherst College, Amherst, MA USA; 2 Department of Philosophy, University of Buffalo, Buffalo, NY USA; 3 Bioethics Research Library, Kennedy Institute of Ethics, Georgetown University, Washington, DC USA; 4 Neuroethics Studies Program, Pellegrino Center for Clinical Bioethics, and Department of Neurology, Georgetown University Medical Center, Washington, DC USA

After the publication of this article [1] it has come to our attention that the following quote from the article by Lombera and Illes [2] “…must have the power — defined by quality of knowledge and ease of access — to help shape that future.”.. was missing a reference citation in the introduction.

The omission of the reference citation for this quote was the result of an error during the production process, and not an omission by, and/or error of the authors.
